# Role of estrogen related receptor beta (*ESRRB*) in DFN35B hearing impairment and dental decay

**DOI:** 10.1186/1471-2350-15-81

**Published:** 2014-07-15

**Authors:** Megan L Weber, Hong-Yuan Hsin, Ersan Kalay, Dana Š Brožková, Takehiko Shimizu, Merve Bayram, Kathleen Deeley, Erika C Küchler, Jessalyn Forella, Timothy D Ruff, Vanessa M Trombetta, Regina C Sencak, Michael Hummel, Jessica Briseño-Ruiz, Shankar K Revu, José M Granjeiro, Leonardo S Antunes, Livia A Antunes, Fernanda V Abreu, Marcelo C Costa, Patricia N Tannure, Mine Koruyucu, Asli Patir, Fernando A Poletta, Juan C Mereb, Eduardo E Castilla, Iêda M Orioli, Mary L Marazita, Hongjiao Ouyang, Thottala Jayaraman, Figen Seymen, Alexandre R Vieira

**Affiliations:** 1 Department of Oral Biology, 614 Salk Hall, School of Dental Medicine, University of Pittsburgh, 3501 Terrace Street, 15261 Pittsburgh, PA, USA; 2 Department of Medical Biology, School of Medicine, Karadeniz Technical University, Trabzon, Turkey; 3 DNA Laboratory of Child Neurology, Charles University 2nd Medical School and University Hospital Motol, Prague, Czech Republic; 4 Department of Pediatric Dentistry, Nihon University School of Dentistry, Matsudo, Chiba, Japan; 5 Department of Pedodontics, Medipol Istanbul University, Istanbul, Turkey; 6 Clinical Research Unit, Fluminense Federal University, Niterói, RJ, Brazil; 7 National Institute of Metrology, Quality and Technology (INMETRO), Duque de Caxias, RJ, Brazil; 8 Department of Specific Formation, School of Dentistry, Fluminense Federal University, Nova Friburgo, RJ, Brazil; 9 Department of Pediatric Dentistry and Orthodontics, Federal University of Rio de Janeiro, Rio de Janeiro, RJ, Brazil; 10 Veiga de Almeida University, Rio de Janeiro, RJ, Brazil; 11 Discipline of Cariology, School of Dentistry, Salgado de Oliveira University, Niterói, RJ, Brazil; 12 Department of Pedodontics, Istanbul University, Istanbul, Turkey; 13 ECLAMC (Latin American Collaborative Study of Congenital Malformations) at CEMIC (Center for Medical Education and Clinical Research), Buenos Aires, Argentina; 14 ECLAMC at INAGEMP-CNPq (National Institute of Population Medical Genetics) at Department of Genetics, Oswaldo Cruz Foundation, Rio de Janeiro, Brazil; 15 ECLAMC at Hospital de Area, El Bolson, RN, Argentina; 16 ECLAMC at INAGEMP-CNPq (National Institute of Population Medical Genetics) at Department of Genetics, Institute of Biology, Center of Health Sciences, Federal University of Rio de Janeiro, Rio de Janeiro, RJ, Brazil; 17 Department of Comprehensive Care, Restorative Dentistry and Endodontics, School of Dental Medicine, the University of Pittsburgh, Pittsburgh, PA, USA; 18 Department of Microbiology and Molecular Genetics, School of Medicine, The University of Pittsburgh, Pittsburgh, PA, USA

**Keywords:** Dental caries, Deafness, Dental development, Ear development, Linkage disequilibrium, Genetics, Polymorphisms

## Abstract

**Background:**

Congenital forms of hearing impairment can be caused by mutations in the estrogen related receptor beta (*ESRRB*) gene. Our initial linkage studies suggested the ESRRB locus is linked to high caries experience in humans.

**Methods:**

We tested for association between the *ESRRB* locus and dental caries in 1,731 subjects, if *ESRRB* was expressed in whole saliva, if *ESRRB* was associated with the microhardness of the dental enamel, and if *ESRRB* was expressed during enamel development of mice.

**Results:**

Two families with recessive *ESRRB* mutations and DFNB35 hearing impairment showed more extensive dental destruction by caries. Expression levels of *ESRRB* in whole saliva samples showed differences depending on sex and dental caries experience.

**Conclusions:**

The common etiology of dental caries and hearing impairment provides a venue to assist in the identification of individuals at risk to either condition and provides options for the development of new caries prevention strategies, if the associated *ESRRB* genetic variants are correlated with efficacy.

## Background

Dental caries is a major public health problem and is estimated to affect 60 to 90 percent of school children as well as a vast number of adults [1]. Also, data from across the world show that children with hearing disorders suffer from poor oral health [2-12]. Congenital forms of hearing impairment can be caused by mutations in the estrogen related receptor beta (*ESRRB*) gene [13-16]. *ESRRB* is located in the 14q24.3 locus, which was linked to dental caries through a genome-wide linkage scan [17]. This is not the first time that hearing loss is associated with alterations of dental structures. Distinct mutations of the dentin sialophosphoprotein gene (*DSPP*), a gene involved in the initial mineralization of the dentin matrix, are responsible for the clinical manifestations of dentinogenesis imperfecta 1 with or without autosomal dominant progressive high frequency sensorineural hearing loss (DFNA39) [18]. In addition, a case control study of 572 college age musicians showed that *ESRRB* nonsynomous SNP rs61742642 (P386S) was associated with bilateral notches in their ears and thus suffered from hearing loss due to acoustic overload [19].

**Figure 1:**
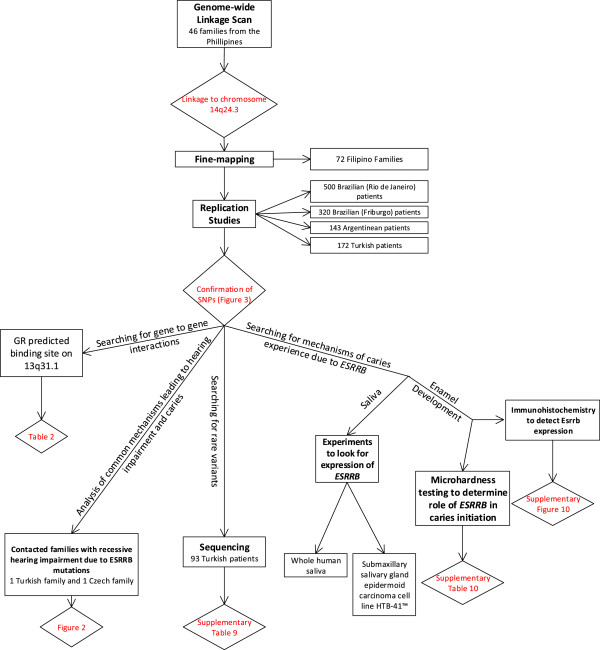
**Flow chart of experiments included in this study.** Experiments listed in order of appearance in manuscript and listed in the boxes. Results are listed in diamonds. Details of each experiment are provided in the Methods section.

Estrogen has a role in the preservation of hearing in aging human adults, and ESRRB binds to estrogen-responsive elements of downstream transcription targets of estrogen signaling [20]. Alterations in transcription, mediated by the glucocorticoid receptor (GR), can contribute to the phenotype of hearing loss related to *ESRRB* mutations because GR, like *ESRRB*, is widely expressed during and after maturation of the mouse and rat cochlea. In addition, ESRRB might repress transcriptional activity mediated by GR [13,20,21]. Here we investigated whether loss of function of *ESRRB*, which in humans leads to hearing impairment, also leads to increased dental caries experience. We used multiple experiments to attempt to gain a thorough idea of how *ESRRB* plays a role in dental decay. Figure 1 provides a visual representation of how the work was conceptualized and developed.

## Methods

**Figure 2:**
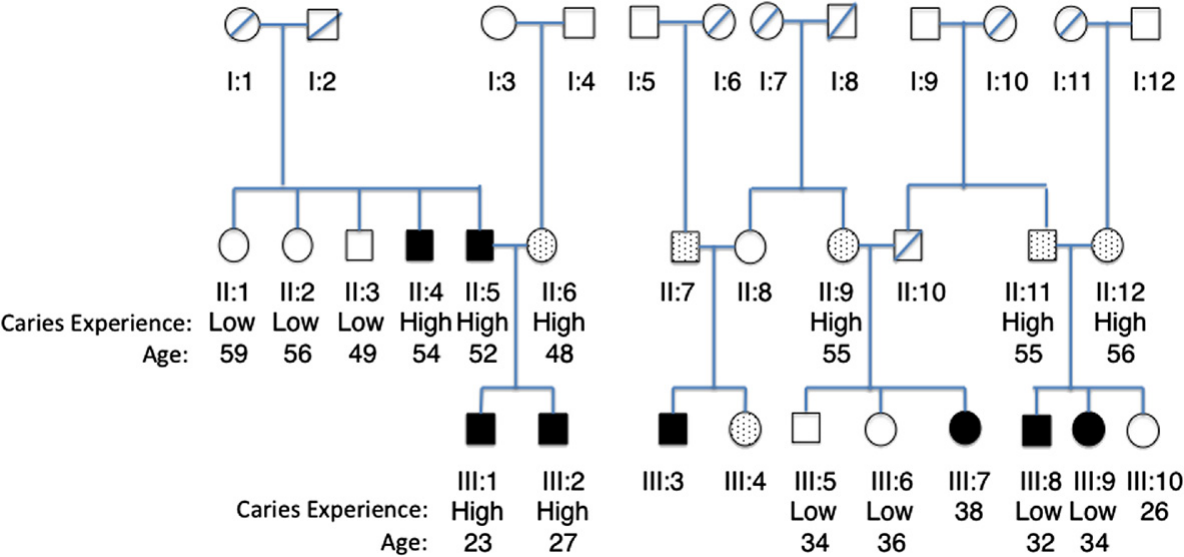
**Dental caries experience of families with hearing impairment DFNB35.** Black indicates recessive hearing impairment. Dots indicate individuals who are heterozygous (carriers) for the mutation. Dental caries levels (high or low) and age in years are indicated below each subject assessed.

Subjects studied are summarized in Table 1. Overall, we studied 1,731 individuals, including two consanguineous families (Figure 2) carrying *ESRRB* mutations associated with congenital hearing loss. Subjects had ancestry in the Philippines, Turkey, Brazil, Argentina, and the Czech Republic. These populations have been described previously [13,16,17,22-26]. The two consanguineous families are from Turkey and the Czech Republic and have been described previously as well [13,16]. The study was approved by the Institutional Review Board of the University of Pittsburgh, appropriate oversight committees of human participation in research in the Philippines, Turkey, Brazil, Argentina, and the Czech Republic, and written informed consent was obtained from each person included in the study. Whole blood was collected for genetic analysis in the cases of the Philippines and the two consanguineous families from Turkey and the Czech Republic. The remaining study groups had DNA purified from whole saliva. Dental caries data was recorded by the use of the Decayed-Missing due to caries-Filled Teeth index (DMFT). Detailed descriptions of the assessments for each population are in the Additional file 1.

**Table 1:** Summary of all individuals analyzed in tests of association, gene expression, and enamel microhardness

	**Filipinos**	**Turkish**	**Brazilian Rio de Janeiro**	**Brazilian Friburgo**	**Argentinean**	**Turkish (Enamel Microhardness)**
Sample size (mean DMFT^a^ ± SD^b^)	477 (9.7 ± 7.3)	172 (3.8 ± 4.0)	500 (2.4 ± 3.0)	320 (1.4 ± 2.7)	143 (7.1 ± 7.8)	100 (5.2 ± 3.4)
High caries group^c^ (mean DMFT ± SD)	298 (13.3 ± 6.7)	92 (7.2 ± 2.3)	171 (5.8 ± 2.6)	53 (6.7 ± 2.8)	66 (13.0 ± 7.9)	63 (7.3 ± 2.5)
Low caries group^c^ (mean DMFT ± SD)	179 (3.6 ± 2.4)	80 (0)	329 (0.6 ± 0.9)	267 (0.4 ± 0.9)	77 (2.0 ± 2.3)	37 (1.7 ± 1.0)
Females	224	93	236	158	83	62
Males	253	79	264	162	60	38
Age (mean ± SD)	25.8 ± 16.3	5.4 ± 0.8	9.1 ± 3.1	3.5 ± 1.5	21.7 ± 15.6	17.2 ± 3.1
The number of pedigrees	72	unrelated	unrelated	unrelated	unrelated	unrelated

^a^Decayed, Missing due to caries, Filled Teeth.

^b^Standard Deviation.

^c^High and low dental caries experience was defined based on our original genome wide linkage study.

Dental caries and tooth loss information was collected for two consanguineous families (one from Turkey [13] segregating a seven base pair duplication mutation and one from the Czech Republic [16] segregating the missense mutation R291L). Families reported on the status of their teeth based on what they were told by their dentist. The detailed reports are presented in Additional file 1: Table S1. Based on these reports, dental caries status was defined as high or low caries experience. These families have recessive hearing impairment due to mutations in *ESRRB*. It is important to note that the family from Turkey comes from a region of low socioeconomic status with limited access to dental care, whereas the family from the Czech Republic resides in a metropolitan area with better access to dental care. Dental data were collected by phone interview (E.K. in Turkey and D.S.B. in the Czech Republic). Of the 17 family members contacted by E.K., 15 provided information regarding their dental caries experience. D.S.B. was able to obtain information from four family members. Dental caries experience between *ESRRB* mutation carriers and non-carriers was compared using the Fisher’s exact test.

To study 14q24.3, single nucleotide polymorphisms (SNPs) were selected using data from the International HapMap project on Caucasians and Chinese (http://www.hapmap.org), which were viewed using Haploview [27]. Twenty-five single nucleotide polymorphisms (SNPs) were selected in 14q24.3 for fine-mapping and are listed in Additional file 1: Table S2 based on pairwise linkage disequilibrium and gene structure data. The Graphical Overview of Linkage Disequilibrium (GOLD) software was used to calculate pairwise linkage disequilibrium between the SNPs and help interpret data [28].

Genotyping was performed using Taqman chemistry end-point analysis. Association between the chosen SNPs and recorded dental caries experience was tested with the transmission disequilibrium test (TDT) implemented in the Family-Based Association Test (FBAT) statistical program [29]. Bonferroni correction was implemented to correct for multiple comparisons and significance was set at 0.002 (0.05/25). Eight SNPs that showed a trend for association with dental caries experience in the Filipino dataset were studied in the additional four population datasets. These eight SNPs were all present within or flanking the *ESRRB* gene. Data were analyzed using the PLINK software [30]. In order to derive a summary statistic for association with the eight SNPs across populations, a random-effects meta-analysis model was used to estimate the odds ratio for the presence of the associated allele determined by the fine-mapping of the Filipino families. Before pooling the data, we estimated Cochran’s Q statistic, which indicates the degree of heterogeneity. There was no significant evidence of heterogeneity overall (Q = 7.0, p = 0.429). A random-effects model was used because it includes variance components both within and between studies. Moreover, because the random-effects model generally yields a wider confidence interval than a fixed-effects model, the random-effects model is more conservative [31].

The less common allele of rs17074565 in 13q31.1 was associated with dental caries and was predicted to disrupt a binding site of GR [32]. Lower expression levels of *GR* in whole saliva are also associated with high dental caries experience [32]. In the Filipino sample, we tested if the eight *ESRRB* SNPs that showed a trend for association with dental caries also interacted with the SNP rs17074565. We observed the transmission of alleles from parents heterozygous for both the rs17074565 SNP and the *ESRRB* SNPs to estimate if specific allele combinations were transmitted more often than expected.

All of the exons and exon-intron boundaries of *ESRRB* were sequenced and compared with the reference sequence transcript ENST00000505752 obtained from Ensembl Genome Browser (http://useast.ensembl.org/index.html). Ninety-three samples from the Turkish cohort were used (62 caries samples and 31 caries free control samples). Primers are listed in Additional file 1: Table S3.

Total RNA isolated from a subset of 94 subjects from the Argentinean population described above was used to test if *ESRRB* expression can be detected in whole saliva. Subsequent cDNA synthesis from 100 ng of total RNA was accomplished by using High Capacity cDNA Reverse Transcription kit (Applied Biosystems). Primers specific for the three *ESRRB* isoforms [13] were tested (*ESRRB* short, long, and Delta10 isoforms listed in Additional file 1: Table S4); *GAPDH* was our endogenous control. Quantitative real-time PCR was performed with SYBR Green PCR Master Mix (Applied Biosystems). Quantification of *ESRRB* expression levels compared to *GAPDH* was performed by 2-DeltaDeltaCT method [33]. Real-time PCR amplification was performed with an initial denaturation at 95°C for five minutes, 60 cycles at 95°C for 45 seconds, 55°C for 45 seconds, and finally 72°C for 90 seconds in a 7900HT Real-time PCR machine. Real-time results were confirmed by western blotting analysis. *ESRRB* expression levels were analyzed based on the presence of zero, one, or two copies of lesser common alleles, sex, and dental caries experience. Non-parametric tests were used in all comparisons.

Total RNA from the submaxillary salivary gland epidermoid carcinoma cell line HTB-41™ (American Type Culture Collection) was isolated and studied. cDNA synthesis and real-time PCR conditions used were described above. *GAPDH* was used as the endogenous control. Amplification of cDNA was performed with SYBR Green PCR Master Mix (Applied Biosystems).

**Figure 3:**
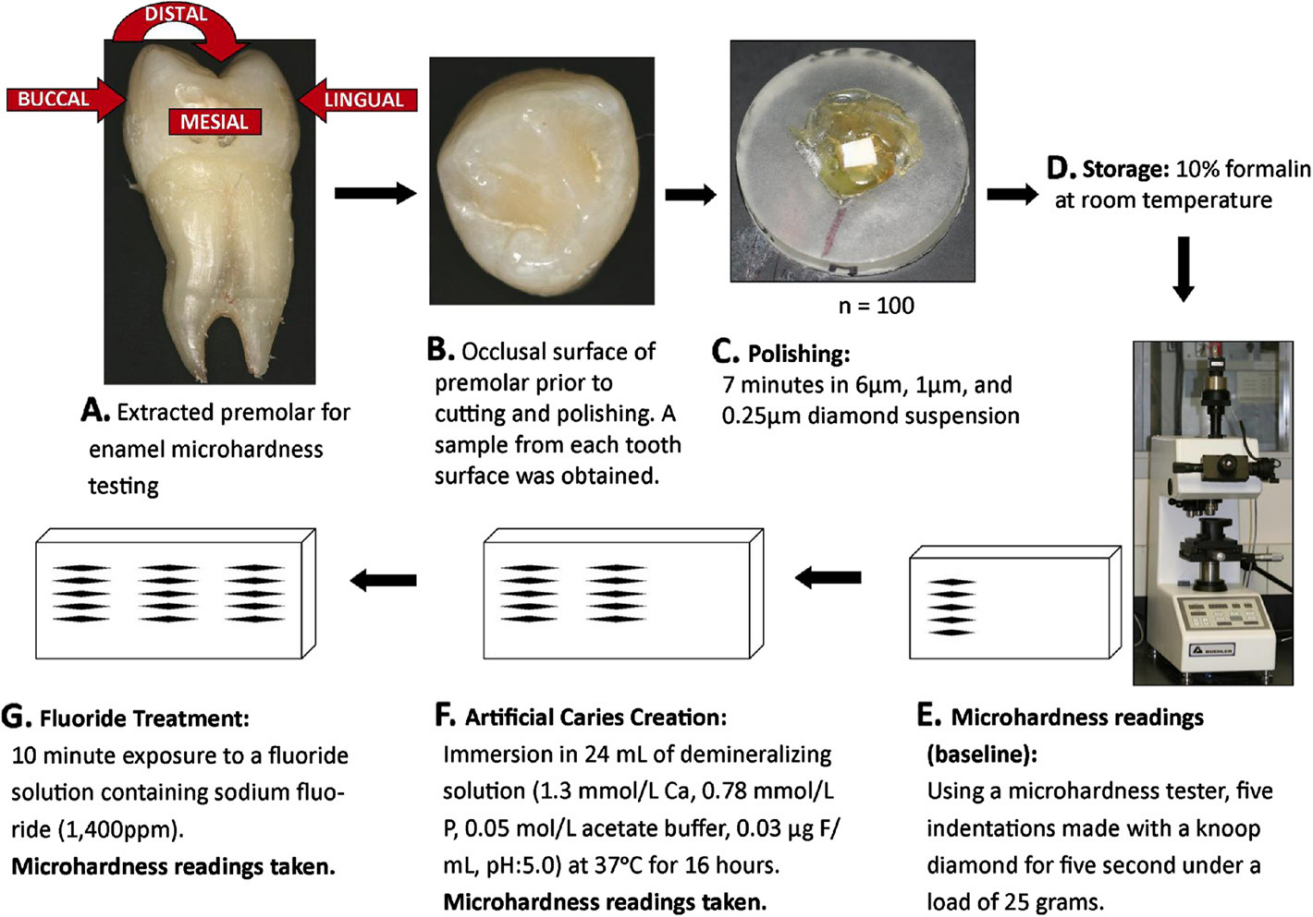
**Enamel microhardness study design.** Image above **A** depicts the five tooth surfaces upon which enamel microhardness was tested. Image above **B** is a close-up of the occlusal surface. Image above **C** shows the appearance of an enamel specimen ready to be tested. Image below **D** shows the testing unit. Microhardness was tested at baseline, after artificial caries creation, and after exposure to a fluoridated solution (panels above **E**, **F**, and **G**. are schematic representations of the assessments).

Enamel samples from extracted premolar teeth from 100 orthodontic adolescent patients (63 with high dental caries experience and 37 with low caries experience, Table 1) from Istanbul University were used in enamel microhardness testing (Figure 3). The enamel samples came from premolars and were used to test the association between genetic variation in *ESRRB* and enamel microhardness at baseline, after simulating artificial caries, and after fluoride treatment. The goal was to test the hypothesis that *ESRRB* influences dental caries by generating a more susceptible enamel surface to acidic dissolution. The Ethics Committee of Istanbul University approved this study, and informed consent from all participating patients was obtained. Subjects age ranged from ten to 32 years (mean age of 17.2 years; 38 males and 62 females). The mean DMFT ranged from zero to 17 (mean DMFT 5.2; 63 with high caries experience and 37 with low caries experience). Tooth samples were cleaned of any remnants and stored in a 10% formalin solution (pH = 7.0) at room temperature until the initial polishing. The crowns of each tooth were separated from the roots and then separated again buccolingually and mesiodistally. The five surfaces studied were occlusal, mesial, buccal, distal, and lingual. The surfaces were sanded for one minute, at a force of 1 lbf, while moving at a speed of 20 rpm on paper of 320, 400, and 600 grit, and then polished for seven minutes at a force of 1 lbf at a speed of 25 rpm in 6 μm, 1 μm, and 0.25 μm diamond suspension. Sample baseline microhardness was tested using a microhardness tester (IndentaMet 1100, Buehler Ltd.) with a knoop diamond. Five indentations under a load of 25 grams for five seconds were made. Next, artificial caries was simulated by immersing the samples in 24 mL of demineralizing solution (1.3 mmol/L Ca, 0.78 mmol/L P, 0.05 mol/L acetate buffer, 0.03 μg F/mL, pH:5.0) at 37°C for 16 hours. Microhardness was again measured by five indentations created just below the initial ones. These indentations were then exposed for ten minutes to a fluoride solution, created from toothpaste containing sodium fluoride (1,400 ppm fluoride), to determine if microhardness for the artificial caries lesions would be brought back to baseline levels after fluoride exposure. Again, surface microhardness was measured with five more indentations below the previous ten.

The results of the microhardness testing were compared to the genotyping of DNA extracted from saliva from each of the 100 patients. Results were analyzed using the PLINK software package^28^. Mean microhardness at baseline, after artificial caries lesion creation, and after fluoride application was calculated. Subjects were divided into two comparison groups: above and below the means. We made comparisons by surface, as well as by using the mean enamel microhardness of all five surfaces combined. A p-value of 0.0004 was considered statistically significant to accommodate for multiple comparisons (Bonferroni correction: 0.05/144).

Expression of Esrrb during enamel development was determined by immunohistochemical analysis of sections of mouse mandibular molars at postnatal day four (secretory stage) and postnatal day eleven (maturation stage).

## Results

**Figure 4:**
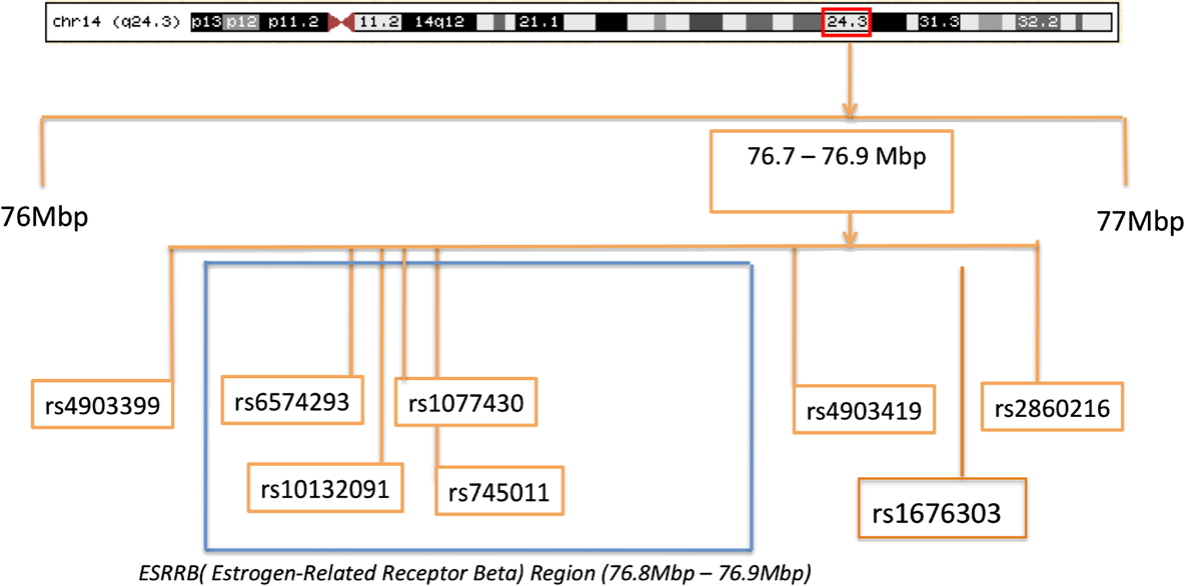
**Summary of fine-mapping results.** At the top, the fine-mapped region is highlighted on 14q24.3. Eight SNPs within or flanking *ESRRB* were associated with high caries experience.

**Figure 5:**
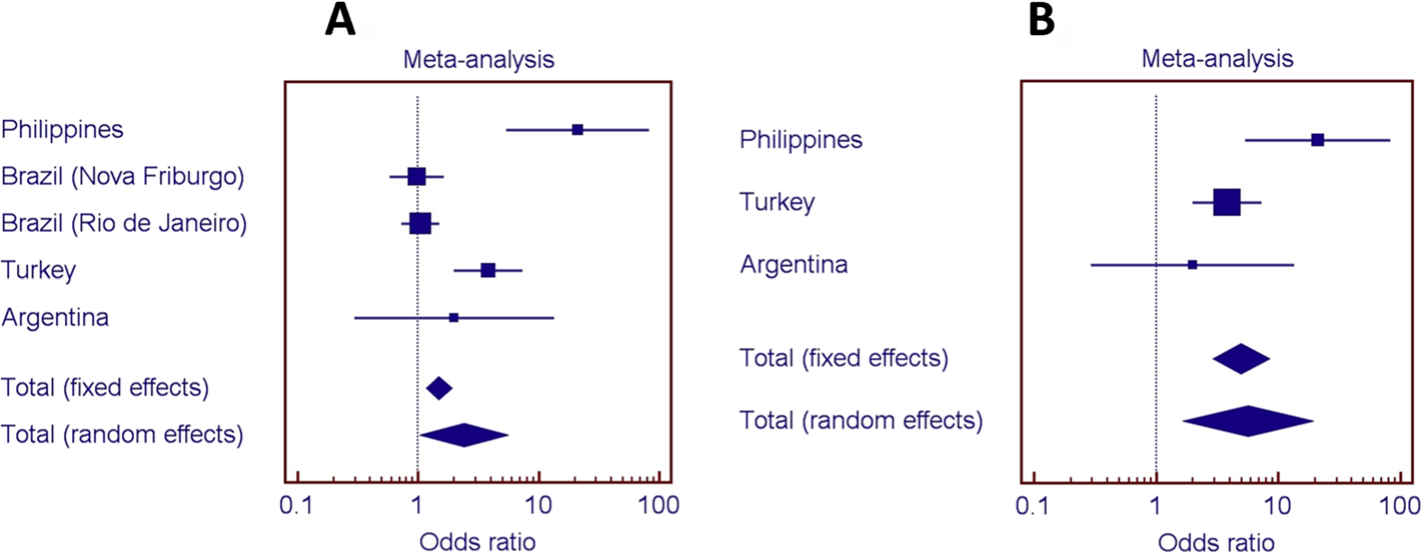
**Odds ratios for over-representation of the T allele (rs1676303) in individuals with high dental caries. A**. Meta-analysis with the five studied groups suggest an association between *ESRRB* rs1676303 and dental caries. **B**. When the two study groups with less dental caries are removed from analysis, the association between *ESRRB* rs1676303 and dental caries becomes stronger.

Of the 25 SNPs used for fine-mapping the region 14q24.3, eight SNPs within or flanking *ESRRB* were found to be over-transmitted in a sample population from the Philippines (Figure 4). Linkage disequilibrium was assessed for these markers and is presented graphically as Additional file 1: Figure S1. Studies of additional populations also indicated associations (details in Additional file 1: Tables S5 through S8). Populations are summarized in Table 1. Figure 5 shows the odds ratios for the association of rs1676303 in samples from the Philippines, Turkey, Brazil, and Argentina. We also show odds ratios when the Brazilian samples, which had lower dental caries experience, were excluded from the meta-analysis (meta-analysis of the additional SNPs can be found in Additional file 1: Figures S2 through S8).

Two families with DFNB35 hearing loss and *ESRRB* mutations were contacted for this study, one from Turkey [13] and the other from the Czech Republic [16]. Of the 17 Turkish DFNB35 family members with a recessive seven base pair duplication in exon 8 of *ESRRB* (c.1018_1024dupGAGTTTG) and hearing impairment [13] contacted by phone interview, 15 provided information regarding their dental caries experience (Figure 2). The ten members of the family who are carriers for the *ESRRB* mutation (six homozygous, four heterozygous) have severe dental caries, with many if not all teeth affected by caries. Three of the five individuals without *ESRRB* mutations were caries free, and two of the five had low caries experience (Fisher’s exact test, p = 0.02). The DFNB35 family from the Czech Republic has a recessive missense mutation (R291L) in *ESRRB* and hearing impairment. Both the mother of the affected child and the mother’s father had high dental caries experience. The affected four-year-old child is caries-free in his primary dentition, and his father is apparently affected by periodontal disease. It was not possible to define the father’s dental caries status.

We also found statistical evidence of an interaction between *ESRRB* SNPs and a SNP predicted to disrupt a GR binding site in families with high dental caries experience (Table 2).

**Table 2:** **Statistical evidence of interaction between rs17074565 (a SNP predicted to disrupt a GR binding site) and *ESRRB *** **SNPs in dental caries in Filipinos**

***ESRRB *** **SNP interacting with rs17074565**	**Number of informative families**	**p-value**	**Associated *ESRRB *** **Allele**
rs4903399	27	0.0000002	C
rs6574293	27	0.00003	G
rs1077430	13	0.21	-
rs4903419	20	0.0000004	A
rs2860216	27	0.05	T
rs10132091	20	0.0000001	T
rs1676303	16	0.0004	T
rs745011	11	0.008	T

From the sequencing of *ESRRB* exons and exon-intron boundaries, SNPs rs10132091, rs61742642, rs3813545, rs3829784, rs45533334, rs35544003, rs2361292, and rs55835922 were found in our samples. There is no evidence indicating these SNPs are disease-causing variants. No mutations causing hearing impairment were found. Individuals with dental caries have an over-representation of the T allele of rs55835922 (74% versus 54%; p = 0.01). The SNP rs61742642 is a missense mutation (P386S), but its frequency was just slightly elevated in cases with dental caries (13% versus 9.5%). SNP rs35544003 is a synonymous change not thought to have any detrimental effect. Detailed sequencing results are listed in Additional file 1: Table S9.

Only expression of the short *ESRRB* isoform listed in Additional file 1: Table S4 was detected, both by real time PCR and western blot analyses (Additional file 1: Figure S9). Additionally, expression of the short *ESRRB* isoform was detected in the submaxillary salivary gland epidermoid carcinoma cell line HTB-41™. The data from the real time PCR experiments indicate that adult females express *ESRRB* in whole saliva in higher levels than men (p = 0.01). Furthermore, a statistical association was found between *ESRRB* expression in whole saliva of children and rs745011 allele distribution (p = 0.04). In a dominant model, statistical association was found between *ESRRB* expression and rs10132091 genotypes (p = 0.03), between low dental caries experience and rs10132091 genotypes (p = 0.05), and between low *ESRRB* expression in whole saliva of adults and rs6574293 genotypes (p = 0.04). In a recessive model, statistical association was found between low *ESRRB* expression and rs2860216 genotypes (p = 0.02).

Through experiments that tested the effects of acid dissolution of the enamel surface, we reasoned that *ESRRB* variants contributes to formation of an enamel structure that is more susceptible to the acidic effects involved in the initiation of dental caries. Distribution of alleles of SNP rs4903419 was different between subjects with harder and softer enamel at baseline under a recessive model (The G allele was associated with harder enamel, p = 0.0007). Also, the distribution of alleles of SNP rs6574293 was different when enamel microhardness at the distal surface was compared after the creation of an artificial caries lesion and after fluoride application (The A allele was associated with harder enamel after fluoride application, p = 0.0006). Complete results are summarized in Additional file 1: Table S10.

Immunohistochemical stain with rabbit polyclonal antibody, demonstrated that Esrrb is expressed by mouse ameloblasts, the cells that deposit tooth enamel, during the secretory stage of amelogenesis in mice (postnatal day four, Additional file 1: Figure S10), but not during the later maturation stage of dental development, such as postnatal day eleven.

## Discussion

Mice that are Esrrb-deficient, or which have a conditional knockout of the *Esrrb* gene, exhibit head-tossing, head-bobbing, and running in circles caused by inner-ear defects [34,35]. In humans, the *ESRRB* autosomal recessive hearing impairment indicates that *ESRRB* is essential for inner-ear development [13-16]. We showed that SNPs in the *ESRRB* locus are also associated with dental caries experience in multiple populations, but particular populations are less influenced by factors that protect against the disease. Results were clearer when we removed the “healthier” groups from the pooled analysis (Figure 5), leaving the ones with limited access to dental care, similar cultural and social behaviors, and sub-optimal exposure to fluoridated drinking water. When we evaluated dental caries in two families with previously described hearing impairment and *ESRRB* mutations, the evidence clearly showed that the severity of dental destruction was much more apparent in mutation carriers. Upon testing the enamel of human teeth in regards to genetic variation in *ESRRB*, we found evidence that “softer” enamel is associated with *ESRRB* SNPs.

ESRRB is suggested to repress transcriptional activity mediated by GR, and these two proteins are widely expressed during and after maturation of the mouse and rat cochlea [13]. We previously showed that a SNP in 13q31.1 (rs17074565) was associated with dental caries and potentially disrupts the GR binding site [32]. We found statistical evidence that rs17074565 and *ESRRB* SNPs are over-transmitted together in families with high dental caries experience.

Both clinical and archeological evidence suggest that women have higher levels of dental caries [36-43], although these differences are not evident when studies are performed in individuals with similar socioeconomic levels and environments [44-46]. The differences are suggested to be the consequence of sex disparities and bias related to the risk factors modulating dental caries [47]. On the other hand, men appear more commonly to have faster hearing deterioration, in part due to the types of occupations that favor males [48]. This evidence is promising in the sense that *ESRRB* detection in whole saliva can be explored not only in regards to risks of dental caries, but also related to risks of hearing loss related to aging or occupational hazard (*i.e.*, dentists [49]). The rationale for this suggestion comes from the hypothesis that *ESRRB* could cause congenital forms of hearing impairment as well as increased susceptibility to the acquired forms of hearing loss. A similar phenomenon happens with diabetes. Data on susceptibility genes and familial clustering for Type 1 and Type 2 Diabetes in humans, mice, and rats suggest the possibility of shared genetic susceptibility to both Type 1 and Type 2 Diabetes in humans [50,51].

We have demonstrated that informed candidate-gene selection aids in identifying specific variants with a role in complex traits that may be otherwise missed by genome-wide association studies [52-55]. The association of dental caries and hearing impairment provides a venue to assist in the identification of individuals at risk to either condition and provides options for the development of new strategies of prevention for both caries and hearing loss, if the associated *ESRRB* genetic variants are correlated with efficacy.

## Conclusions

*ESRRB*, a gene when mutated causes a form of hearing impairment, also contributes to dental decay likely by influencing the formation of an enamel surface more susceptible to demineralization under acidic conditions.

## Web resources

The URLs for presented data are as follows:

dbSNP: http://www.ncbi.nlm.nih.gov/projects/SNP.

Ensemble Genome Browser: http://www.ensembl.org/index.html.

HapMap Project: http://hapmap.ncbi.nlm.nih.gov.

PLINK: http://pngu.mgh.harvard.edu/~purcell/plink/.

Primer3: http://biotools.umassmed.edu/bioapps/primer3_www.cgi.

UCSC Genome Bioinformatics: http://genomebrowser.ucsc.edu.

## Competing interests

The authors have no competing interests to declare.

## Authors’ contributions

MLW and ARV wrote the manuscript. All authors critically revised and approved the version submitted for publication. MLW, H-YH, EK, DSB, TS, MB, KD, ECC, JF, TDR, VMT, RCS, MH, JB-R, SKR, HO, and TJ generated and analyzed data and helped interpret results. JMG, LSA, LAA, FVA, MCC, and PNT collected clinical and biological patient data at the Brazilian site. JMG. ECK, and MCC helped design the component of this study developed in Brazil. EK, MK, AP, and MB collected clinical and biological patient data at the Turkish site. FS and EK helped design the component of this study developed in Turkey. FAP, JCM, IMO, MLM, and ECC helped design the component of this study developed in Argentina. FAP, ARV, and JB-R collected clinical and biological patient data at the Argentinean sites. ARV designed and provided oversight on all components of this project. All authors read and approved the final manuscript.

## Pre-publication history

The pre-publication history for this paper can be accessed here:

http://www.biomedcentral.com/1471-2350/15/81/prepub

## Supplementary Material

Additional file 1: Figure S1 Linkage Disequilibrium (D’) of *ESRRB* SNPs Showing Association with Dental Caries. **Figure S2.** Results of Meta-Analyses (rs745011). **Figure S3.** Results of Meta-Analyses (rs1077430). **Figure S4.** Results of Meta-Analyses (rs2860216). **Figure S5.** Results of Meta-Analyses (rs4903399). **Figure S6.** Results of Meta-Analyses (rs4903419). **Figure S7.** Results of Meta-Analyses (rs6574293). **Figure S8.** Results of Meta-Analyses (rs10132091). **Figure S9.** Western Blot of ESRRB in Whole Saliva from 10 Healthy Female Subjects. **Figure S10.** Immunohistochemical analysis showing Esrrb expression in secretory stage ameloblasts. **Table S1.** Dental Caries Status of the Two Families Segregating *ESRRB* Mutations and DFNB35. **Table S2.** SNPs Studied and Summary of Results of Fine-Mapping of the Filipino Population. **Table S3.** Primer Sets for Sequencing *ESRRB* exons and exon-intron boundaries. used for polymerase chain reaction (PCR) amplification were designed using Primer3 software [56] and supplied by Integrated DNA Technologies (Integrated DNA Technologies, Inc.). The samples were sent to Functional Biosciences, Inc. for purification and sequencing. The sequences were then verified against the reference sequence transcript and the sequences from two unrelated CEPH (Foundation Jean Dausset-Centre d’Etude du Polymorphisme Humain) DNA samples [57] using Sequencher 5.1 software (Gene Codes Corporation). **Table S4.** Primer Sets and Detailed Methods for RT-PCR Experiments. **Table S5.** Results of Association Studies in the Turkish Children. **Table S6.** Results of Association Studies in the Brazilian (Nova Friburgo) Children. **Table S7.** Results of Association Studies in the Brazilian (Rio de Janeiro) Children. **Table S8.** Results of Association Studies in the Argentinean Population. **Table S9.** Summary of Sequencing Results and Case-Control Comparisons. **Table S10.** Workflow and Results of Enamel Microhardness Testing [1,17,20,24,55,56,58]. Click here for file
