# Supplementary material for: Role of estrogen related receptor beta (ESRRB) in DFN35B hearing impairment and dental decay
Source: BMC Med Genet. 2014 Jul 15;15:81. doi: 10.1186/1471-2350-15-81 (PMC4112727; doi:10.1186/1471-2350-15-81)
Supplement: Additional file 1: Figure S1 — Linkage Disequilibrium (D’) of ESRRB SNPs Showing Association with Dental Caries. Figure S2. Results of Meta-Analyses (rs745011). Figure S3. Results of Meta-Analyses (rs1077430). Figure S4. Results of Meta-Analyses (rs2860216). Figure S5. Results of Meta-Analyses (rs4903399). Figure S6. Results of Meta-Analyses (rs4903419). Figure S7. Results of Meta-Analyses (rs6574293). Figure S8. Results of Meta-Analyses (rs10132091). Figure S9. Western Blot of ESRRB in Whole Saliva from 10 Healthy Female Subjects. Figure S10. Immunohistochemical analysis showing Esrrb expression in secretory stage ameloblasts. Table S1. Dental Caries Status of the Two Families Segregating ESRRB Mutations and DFNB35. Table S2. SNPs Studied and Summary of Results of Fine-Mapping of the Filipino Population. Table S3. Primer Sets for Sequencing ESRRB exons and exon-intron boundaries. used for polymerase chain reaction (PCR) amplification were designed using Primer3 software [56] and supplied by Integrated DNA Technologies (Integrated DNA Technologies, Inc.). The samples were sent to Functional Biosciences, Inc. for purification and sequencing. The sequences were then verified against the reference sequence transcript and the sequences from two unrelated CEPH (Foundation Jean Dausset-Centre d’Etude du Polymorphisme Humain) DNA samples [57] using Sequencher 5.1 software (Gene Codes Corporation). Table S4. Primer Sets and Detailed Methods for RT-PCR Experiments. Table S5. Results of Association Studies in the Turkish Children. Table S6. Results of Association Studies in the Brazilian (Nova Friburgo) Children. Table S7. Results of Association Studies in the Brazilian (Rio de Janeiro) Children. Table S8. Results of Association Studies in the Argentinean Population. Table S9. Summary of Sequencing Results and Case-Control Comparisons. Table S10. Workflow and Results of Enamel Microhardness Testing [1,17,20,24,55,56,58]. [file 1471-2350-15-81-S1.docx]

**Supplemental Appendix**

**Detailed Population Assessments**

**The Philippines**

Study site, subject recruitment, and DMFT score collection for the Filipino samples were those used in our prior genome-wide linkage study [17]. The 477 subjects (253 males and 224 females) from 72 pedigrees came mainly from Cebu Island and share a common Malaysian ancestry. This allowed us to reduce the influence of behavioral and environmental factors on caries experience. DNA samples were extracted from peripheral blood according to standard protocols. The age range of the subjects was two to 72 years (mean age 22.6 years). The mean DMFT score was 9.7 with a range of zero to 32. Dental caries experience definitions for fine-mapping and all follow-up studies were made according to our original genome-wide linkage analysis [17]. The University of Pittsburgh and the H.O.P.E Foundation International Institutional Review Boards approved the study protocol. Also, the appropriate informed, written consent was obtained from all participants, and age appropriate assent documents were used for children seven to 14 years of age. This allowed us to obtain informed, written consent from both the child and the parents. DMFT scores were collected by one calibrated examiner (A.R.V.). No intra-examiner data could be generated.

**Follow-up Studies**

The samples for each replication study are outlined in Table 1. Samples from Istanbul, Turkey included 172 patients (79 males and 93 females), who had a mean age of 5.4 years with a range of three to six years of age. Ninety-two of these patients had high caries experience with a dmft (caries experience in the primary dentition) of four or more, and 80 of the children were caries-free [22]. Istanbul University and the University of Pittsburgh Institutional Review Boards approved the study of these samples, and appropriate written informed consent was obtained from the parents of all participating children.

The samples from Rio de Janeiro, Brazil included 500 patients (264 males and 236 females). These patients had a mean age of nine years with a range of seven to 14 years. Samples were used with the approval of the University of Pittsburgh Institutional Review Board and the Federal University of Rio de Janeiro Institutional Review Board. Age appropriate assent documents were used for children between seven and 14 years, and informed written consent was obtained from both the child and the parents.

A study sample from Nova Friburgo, Brazil was used as well. This included 320 subjects (162 males and 158 females). The children had a mean age of 3.5 with a range of one to six years. The mean dmft score was 1.4 with a range of zero to 16. Samples were used with the approval of the University of Pittsburgh Institutional Review Board and the Fluminense Federal University ethics committee.

Samples from subjects recruited in the Patagonian Argentina were also used. Samples from a subset of these subjects were also used in the real-time PCR experiments. Sixty-six subjects had a mean age of 27.96 years, ranging from two to 72 years old. Seventy-seven had low caries experience as determined by DMFT/dmft scores stratified by age, and 68 had high caries experience. Samples were used with the approval of the University of Pittsburgh Institutional Review Board and the Centro de Educación Médica e Investigaciones Clínicas “Norberto Quirno” (CEMIC).

Dental caries was diagnosed using a modified World Health Organization protocol recommended for oral health surveys [1]. Teeth lost to trauma or primary teeth lost to exfoliation were not included in the final DMFT/dmft scores. When records indicated that teeth were extracted for orthodontic reasons or periodontal disease, or treatments were performed in sound teeth, these situations were not included in the final DMFT/dmft scores. The studies developed in Turkey included white spot lesions as evidence of caries. For all studies, carious lesions were recorded as present when a break in enamel was apparent on visual inspection. All the examiners carried out the clinical examination after being calibrated by an experienced specialist. Details about the determination of caries experience were previously described [22,24].

In this study, the populations were classified as either ‘low caries experience’ or ‘high caries experience,’ based on DMFT/dmft distribution in each cohort (DMFT/dmft mean and standard deviation) and the subject’s age. The criteria used here for classification of caries experience took age into consideration, since it is expected that caries experience will increase in the general population with age [56].

**Supplemental Figure 1. Linkage Disequilibrium (D’) of *ESRRB* SNPs Showing Association with Dental Caries (**1**=**rs2860216, 2=rs4903419, 3=rs745011, 4=rs1077430, 5=rs10132091, 6=rs6574293, and 7=rs4903399).

**
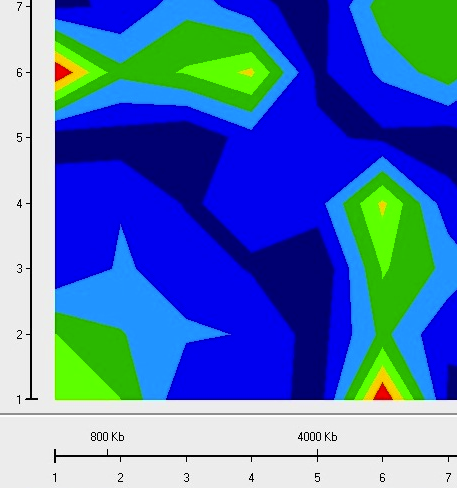
**

**Supplemental Figure 2. Results of Meta-Analyses.**

**rs745011**

**
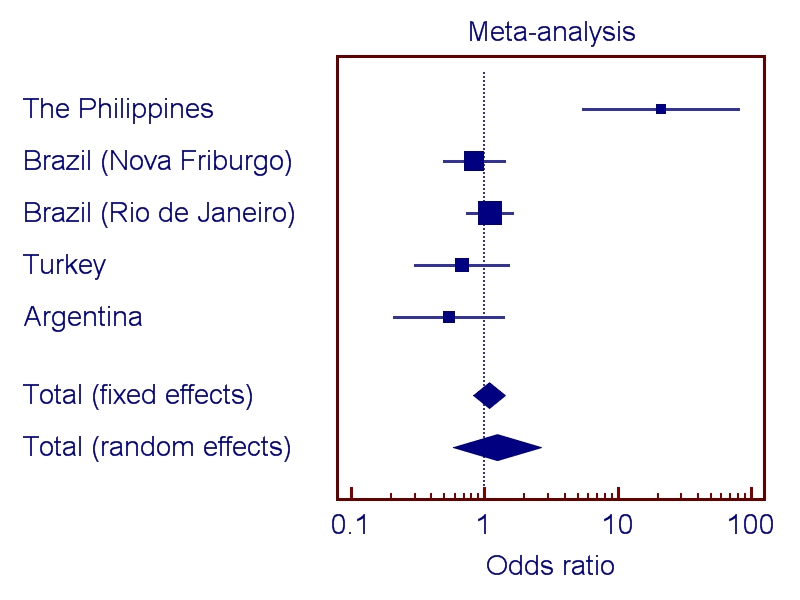
**

**Supplemental Figure 3. Results of Meta-Analyses.**

**rs1077430**

**
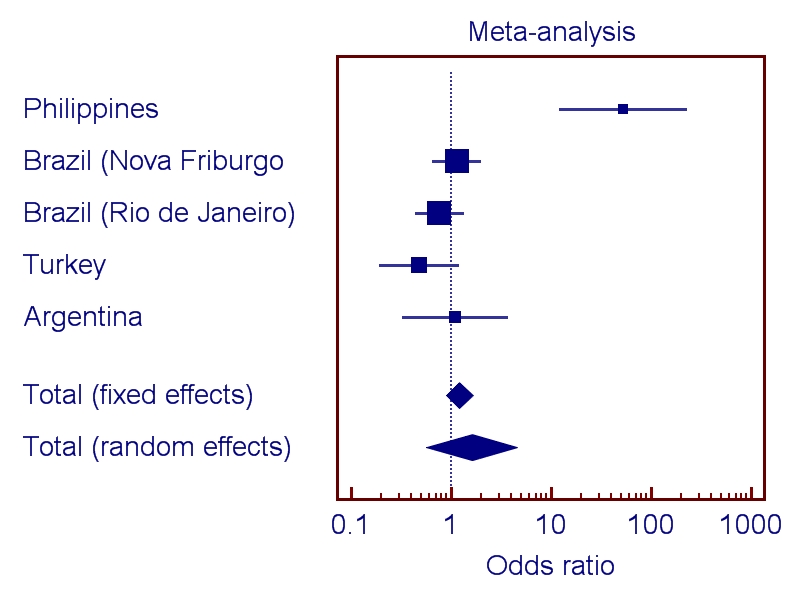
**

**Supplemental Figure 4. Results of Meta-Analyses.**

**rs2860216**

**
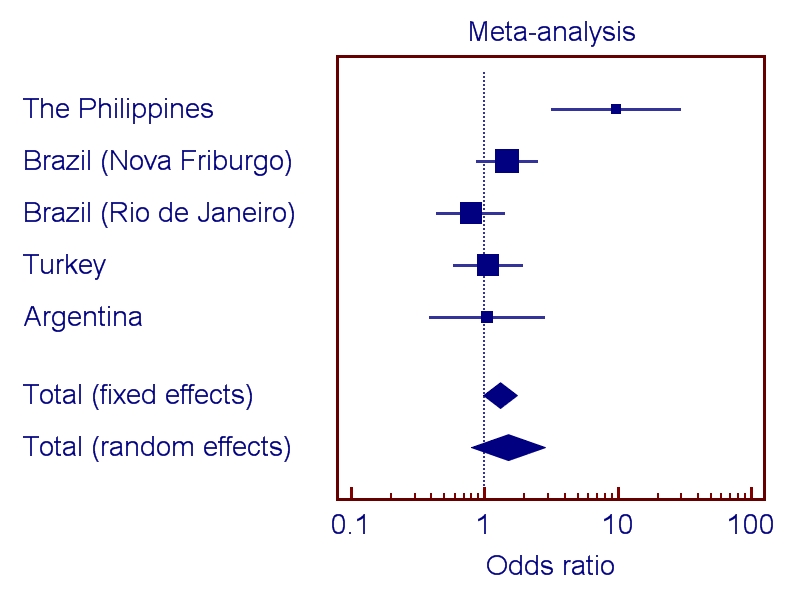
**

**Supplemental Figure 5. Results of Meta-Analyses.**

**rs4903399**

**
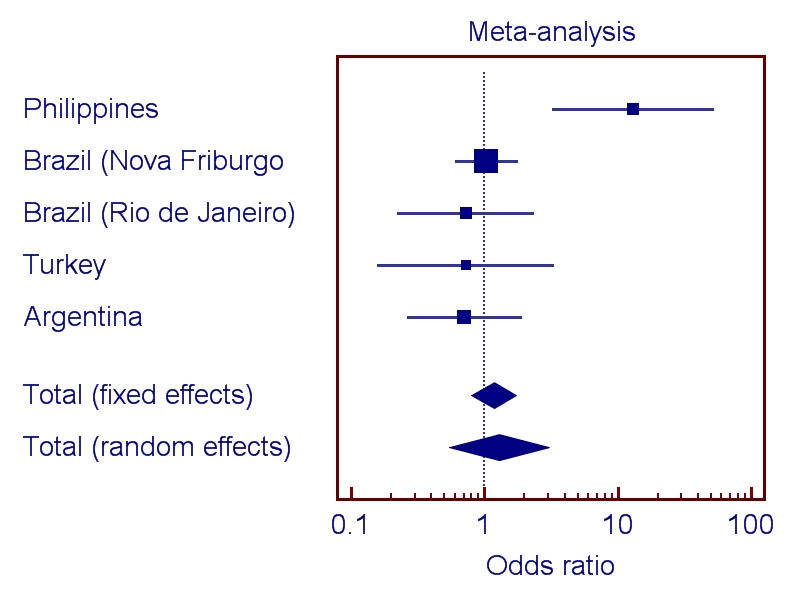
**

**Supplemental Figure 6. Results of Meta-Analyses.**

**rs4903419**

**
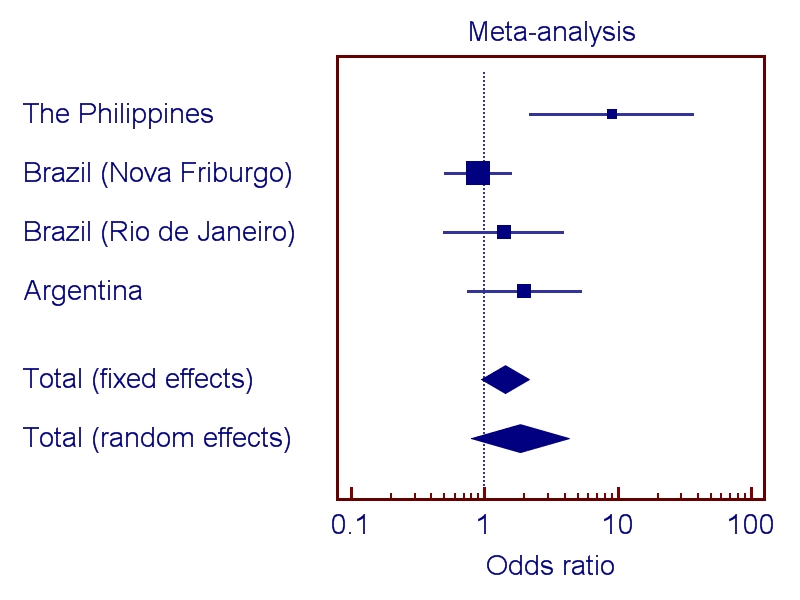
**

**Supplemental Figure 7. Results of Meta-Analyses.**

**rs6574293**

**
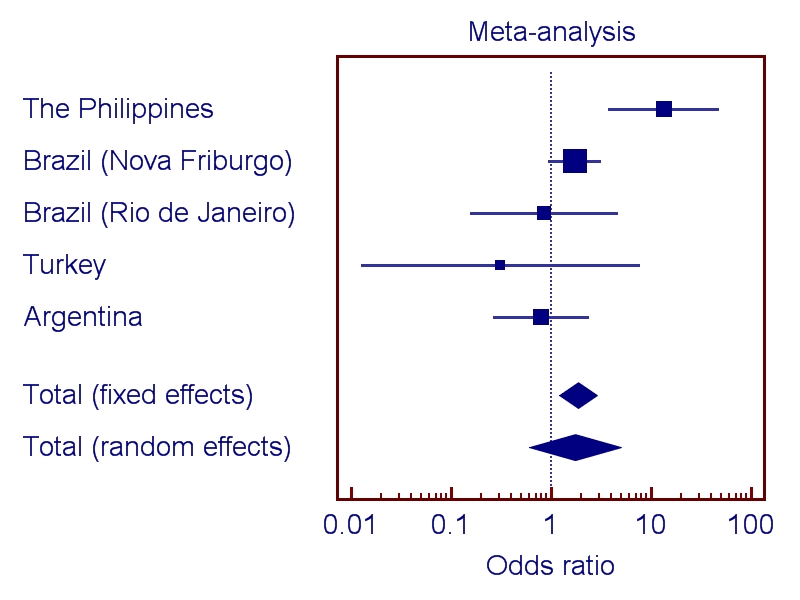
**

**Supplemental Figure 8. Results of Meta-Analyses.**

**rs10132091**

**
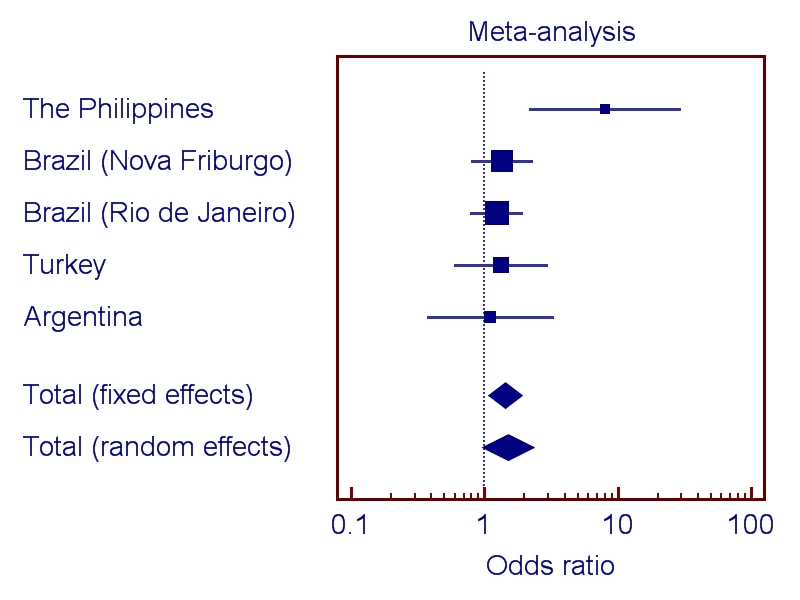
**


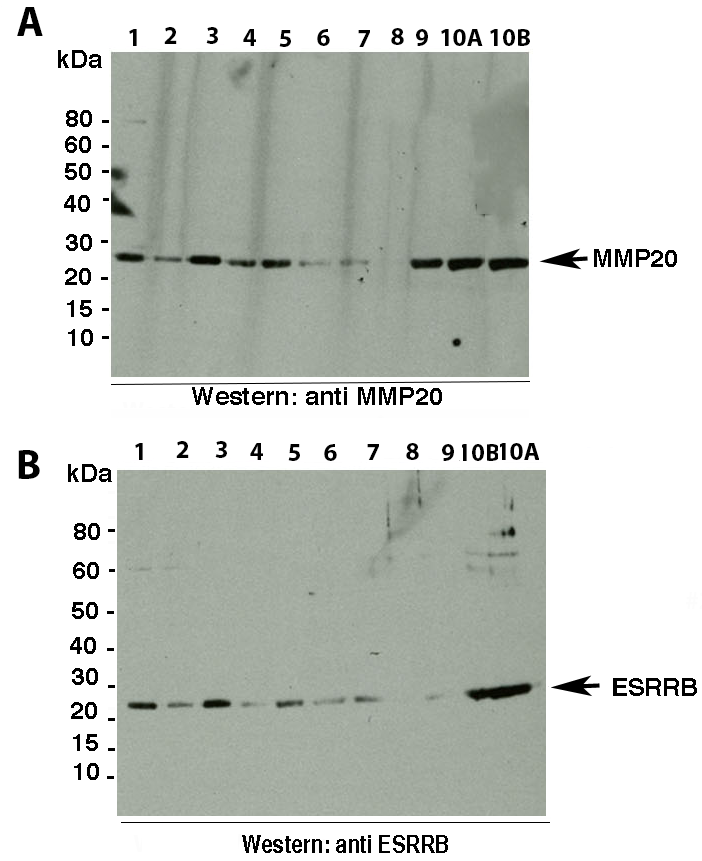
**Supplemental Figure 9. Western Blot analysis of ESRRB in Whole Saliva from 10 Healthy Female Subjects.** Subject 10 (A and B are two samples from the same individual) had active dental work done which may relate to the higher levels of ESRRB. Quantitative inferences can be made from known equal amounts of protein concentrations loaded into the gel (17.5 μg per lane). Since we were working with saliva samples, we did not use house keeping gene product antibodies as they are not secreted proteins.

**Supplemental Figure 10. Immunohistochemical Analysis Showing Esrrb Expression in Secretory Stage Ameloblasts.** Immunohistochemistry of demineralized, paraffin sections of mouse mandibular molar at 4 days postnatal (20X).

**
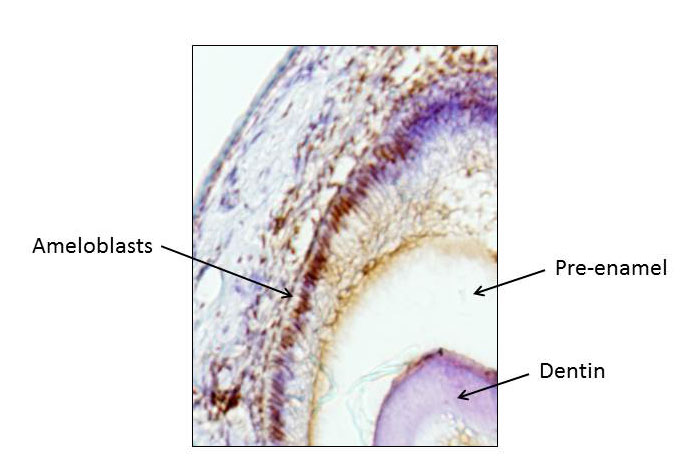
**

**Supplemental Table 1. Dental Caries Status of the Two Families Segregating *ESRRB* Mutations and DFNB35.**

**DFNB35 Turkish Family**

| Individual | Age (Years) | ESRRB Mutation Status | Self-Report | Dental Caries Experience Definition |
| --- | --- | --- | --- | --- |
| II:6 | 48 | Heterozygous | Edentulous since 3 years ago due to caries and gum recession | High |
| II:5 | 52 | Homozygous | Edentulous since 5 years ago due to caries and gum recession | High |
| II:4 | 54 | Homozygous | Edentulous due to caries and gum recession | High |
| II:3 | 49 | Not a carrier | No problems reported | Low |
| II:2 | 56 | Not a carrier | No problems reported | Low |
| II:1 | 59 | Not a carrier | No problems reported | Low |
| III:1 | 23 | Homozygous | Edentulous on the maxillary arch and posterior mandibular | High |
| III:2 | 27 | Homozygous | Molars with decay | High |
| II:11 | 55 | Heterozygous | All teeth except 10 extracted by the person since they were mobile. Last 10 teeth extracted by a dentist more recently (caries and gum recession) | High |
| **Supplemental Table 1. Dental Caries Status of the Two Families Segregating *ESRRB* Mutations and DFNB35 continued.** | | | | |
| II:12 | 56 | Heterozygous | Edentulous since 5 years ago due to caries and gum recession | High |
| III:8 | 32 | Homozygous | Two molars extracted due to pain and inflammation | Low |
| III:9 | 34 | Homozygous | 3 to 5 teeth extracted | Low |
| III:10 | 26 | Unknown | Unknown |  |
| II:9 | 55 | Heterozygous | All maxillary teeth with the exception of the four incisors were extracted, as well as all mandibular teeth (caries and every pregnancy 2 or 3 teeth were extracted due to pain) | High |
| III:6 | 36 | Unknown | 3 teeth extracted and 2 teeth with fillings (caries) | Low |
| III:5 | 34 | Unknown | 6 teeth extracted and 2 or 3 teeth with fillings (caries) | Low |
| III:7 | 38 | Homozygous | Unknown |  |

**DFNB35 Czech Family**

| Individual | Age (Years) | *ESRRB* Mutation Status | Self-Report | Dental Caries Experience Definition |
| --- | --- | --- | --- | --- |
| III:3 | 4 | Homozygous | Caries-free | Low |
| **Supplemental Table 1. Dental Caries Status of the Two Families Segregating *ESRRB* Mutations and DFNB35 continued.** | | | | |
| II:1 | 32 | Heterozygous | Periodontitis, and increased “dental stones” | Unknown |
| II:2 | 32 | Heterozygous | Increased caries after age of 24 (7 new cavities), which started before pregnancy | High |
| I:3 | 55 | Heterozygous (DNA testing was not possible, but due to the consanguinity of the pedigree, this person is likely to be a carrier) | Increased caries after age of 25 | High |

**Supplemental Table 2. SNPs Studied and Summary of Results of Fine-Mapping of the Filipino Population.**

| **Marker** | **Allele** | **Minor Allele Frequency** | **Gene** | **Location** | **p-value** |
| --- | --- | --- | --- | --- | --- |
| rs4903399 | T | 0.28 |  | chr. 14 76775202 | 0.02 |
| rs6574293 | A | 0.07 | *ESRRB* | chr. 14 76870600 | 0.007 |
| rs7160372 | T | 0.45 |  | chr. 14 76816376 | 0.47 |
| rs1642866 | A | 0.15 |  | chr. 14 77014035 | 0.34 |
| rs7151924 | C | 0.31 |  | chr. 14 76778069 | 0.17 |
| rs4903419 | G | 0.11 |  | chr. 14 76984655 | 0.009 |
| rs2361289 | C | 0.20 | *ESRRB* | chr. 14 76910170 | 0.17 |
| rs1077430 | C | 0.30 | *ESRRB* | chr. 14 76897677 | 0.09 |
| rs2860216 | C | 0.29 |  | chr. 14 77006008 | 0.003 |
| rs11159198 | T | 0.45 | *ESRRB* | chr. 14 76867381 | 0.61 |
| rs1642818 | C | 0.35 |  | chr. 14 77057801 | 0.26 |
| rs7160845 | C | 0.20 |  | chr. 14 77038124 | 0.42 |
| rs10132091 | C | 0.42 | *ESRRB* | chr. 14 76870818 | 0.007 |
| rs6574292 | A | 0.39 | *ESRRB* | chr. 14 76866718 | 0.46 |
| rs11623206 | C | 0.28 |  | chr. 14 76750175 | 0.56 |
| rs9944037 | C | 0.41 | *ESRRB* | chr. 14 76903733 | 0.92 |
| rs12586444 | T | 0.48 | *ESRRB* | chr. 14 76861565 | 0.91 |
| rs6574310 | A | 0.19 |  | chr. 14 77049704 | 0.69 |
| rs1676303 | C | 0.13 |  | chr. 14 76992164 | 0.06 |
| rs1867654 | A | 0.43 | *ESRRB* | chr. 14 76937722 | 0.53 |
| rs7152058 | T | 0.20 |  | chr. 14 76806538 | 0.91 |
| rs12437118 | A | 0.37 |  | chr. 14 76983730 | 0.31 |
| rs745011 | C | 0.33 | *ESRRB* | chr. 14 76917275 | 0.04 |
| rs9323631 | T | 0.35 | *ESRRB* | chr. 14 76951278 | 0.1 |
| rs2885232 | T | 0.35 | *ESRRB* | chr. 14 76957391 | 0.52 |
| rs958729 | T | 0.12 |  | chr. 14 76991986 | 0.8 |

**Supplemental Table 3. Primer Sets for Sequencing *ESRRB* exons and exon-intron boundaries.**

| **Gene Exon** | **Forward Primer (5’-3’)** | **Reverse Primer (5’-3’)** | **Temperature** | **Product Size (Base Pairs)** |
| --- | --- | --- | --- | --- |
| 1 | ACTTTCCTGCGTCCATCAGT | CAGAGTCTGGGGAGGAGAAA | 56.5 | 378 |
| 2 | ATGTTTCCGCAGCATTTATC | GCCACATGCTCTCTAAATCC | 51.3 | 578 |
| 3 | CTCCTCCCACTCTGCGTTC | GGACAGACAGACCGAGAAGC | 57.7 | 214 |
| 4 | CCACAAACAGTGTGTCTGCAT | GGTCTGGCCATTCATTCATT | 55.9 | 248 |
| 5 | ATGCAATGTGACCCTAGAGC | AAGACAGCATGGTCTGCATC | 54.9 | 337 |
| 6 | TAATGCCAGAAACTTGCTCC | CACAGAAGTACCGCTCCAAC | 53.0 | 720 |
| 7 | TGCTTTGAGAACACTAGGGG | AAGAAATTCCAATTCCCACC | 54.5 | 448 |
| 8 | CCCTGCGTCCTCTGTCTCTA | AGAGCAAGACTCCGTCTCCA | 58.0 | 430 |
| 9 | GGATGCGCCATTACTGTTAG | CCCAAGATCCACATTGTCTC | 53.6 | 424 |
| 10 | GGAGCTCTTAGGAACCCAAC | TCCTCTCCAATGCTACAAGG | 55.1 | 439 |
| 11 | TTACGCTACACAGGGAAAGC | CTTAGGAAATGCTCAGCCAG | 54.9 | 640 |
| 12 | ACCTCTTGAGAAATGTCCCC | CATGATACAGGGGTTGAAGG | 54.3 | 577 |
| 12 | TGCTAATGCTCGTCCTTGTG | GAGCCATGATACAGGGGTTG | 55.2 | 380 |
| 13 | TGTGGGGCTCGACTGTAACT | CACTCCTGAAGGGAGTCAGC | 58.3 | 98 |
| 14 | TCCCAGGAAACTCCTCTACC | AGCTGCTCTGCAGTTTGTG | 55.6 | 477 |
| 14 | CTCACTGTGCTGTGTCCTTG | ATGGACCCCTTCAGTACCAG | 55.9 | 393 |
| 14 | CTGGCATCTGCTGTCTGTCT | GGAGAGGGTCCTCATCTGG | 57.1 | 365 |
| 14 | AGTTCAGGGGCCAACTTCTT | TGACAAAGCCAGTCTGGAAA | 56.6 | 336 |
| 14 | TCCCCTTGTCCTTATACGTTC | CCAGAAAGACAATGTGTGAAGAA | 53.8 | 600 |

Primers used for polymerase chain reaction (PCR) amplification were designed using Primer3 software [57] and supplied by Integrated DNA Technologies (Integrated DNA Technologies, Inc.). The samples were sent to Functional Biosciences, Inc. for purification and sequencing. The sequences were then verified against the reference sequence transcript and the sequences from two unrelated CEPH (Foundation Jean Dausset-Centre d’Etude du Polymorphisme Humain) DNA samples [58] using Sequencher 5.1 software (Gene Codes Corporation).

**Supplemental Table 4. Primer Sets.**

| **Gene** | **Forward Primer (5’-3’)** | **Reverse Primer (5’-3’)** | **Product Size (Base Pairs)** |
| --- | --- | --- | --- |
| *ESRRBlong* | ctatagcgtcaaactgcagggcaaagtg | ctgctcttggccaacctgccctct | Less than 100 |
| *ESRRBshort* | tgacgacaagctggtgtacg | tctgtaggtgggcattggtc | 511 |
| *ESRRB- Δ10* | tgacgacaagctggtgtacg | atccctgcttgtgaaggcag | 511 |
| *GAPDH* | accacagtccatgccatcac | tccaccaccctgttgctgta | 452 |

| *ESRRB*long, estrogen-related receptor beta long isoform |
| --- |
| *ESRRB*short, estrogen-related receptor beta short isoform |
| *ESRRB*- Δ10, estrogen-related receptor beta Δ10 isoform |
| *GAPDH***,** glyceraldehyde-3-phosphate dehydrogenase |

**Supplemental Table 5. Results of Association Studies in the Turkish Children.**

| Marker | Cases | Controls |
| --- | --- | --- |
| rs4903399 |  |  |
| CC | 52 | 47 |
| CT | 31 | 33 |
| TT | 3 | 4 |
|  |  | p= 0.8 |
| C | 135 | 127 |
| T | 37 | 49 |
|  |  | p=0.17 |
| rs6574293 |  |  |
| AA | 0 | 1 |
| AG | 4 | 6 |
| GG | 85 | 77 |
|  |  | p=0.44 |
| A | 4 | 8 |
| G | 174 | 160 |
|  |  | p=0.2 |
| rs1077430 |  |  |
| CC | 8 | 15 |
| CT | 30 | 33 |
| TT | 49 | 38 |
|  |  | p=0.16 |
| C | 46 | 63 |
| T | 128 | 109 |
|  |  | p=0.04 |
| rs2860216 |  |  |
| CC | 7 | 6 |
| CT | 37 | 36 |
| TT | 41 | 42 |
|  |  | p=0.95 |
| C | 51 | 48 |
| T | 119 | 120 |
|  |  | p=0.77 |
| **Supplemental Table 5. Results of Association Studies in the Turkish Children Continued.** | | |
| rs10132091 |  |  |
| CC | 16 | 12 |
| CT | 45 | 47 |
| TT | 27 | 25 |
|  |  | p=0.74 |
| C | 77 | 71 |
| T | 99 | 97 |
|  |  | p=0.78 |
| rs1676303 |  |  |
| CC | 1 | 14 |
| CT | 21 | 13 |
| TT | 66 | 56 |
|  |  | p=0.001 |
| C | 23 | 41 |
| T | 153 | 125 |
|  |  | p=0.006 |
| rs745011 |  |  |
| CC | 12 | 15 |
| CT | 32 | 31 |
| TT | 44 | 34 |
|  |  | p= 0.53 |
| C | 56 | 61 |
| T | 120 | 99 |
|  |  | p=0.22 |

**Supplemental Table 6. Results of Association Studies in the Brazilian (Nova Friburgo) Children.**

| **rs#** |  | **Genotype frequency (%)** | | | | | | **p-value** | | | |
| --- | --- | --- | --- | --- | --- | --- | --- | --- | --- | --- | --- |
|  | **Allele** | **Affected group** | | | **Unaffected group** | | | **Genotype** | **Allele** | **Dominant Model** | **Recessive Model** |
|  |  | **dd** | **dD** | **DD** | **dd** | **dD** | **DD** |  |  |  |  |
| rs4903399 | G/A | 3 | 29 | 63 | 9 | 43 | 106 | 0.59 | 0.81 | 0.9 | 0.36 |
| rs6547293 | A/G | 4 | 21 | 71 | 9 | 19 | 137 | 0.08 | 0.2 | 0.08 | 0.64 |
| rs1077430 | A/G | 10 | 39 | 32 | 17 | 57 | 55 | 0.85 | 0.81 | 0.65 | 0.86 |
| rs2860216 | A/G | 17 | 45 | 32 | 30 | 59 | 68 | 0.25 | 0.36 | 0.15 | 0.84 |
| rs10132091 | A/G | 25 | 40 | 31 | 225 | 72 | 63 | 0.12 | 0.05 | 0.25 | 0.04 |
| rs1676303 | A/G | 5 | 30 | 64 | 8 | 51 | 110 | 0.99 | 0.91 | 0.94 | 0.91 |
| rs745011 | A/G | 18 | 38 | 33 | 31 | 74 | 52 | 0.77 | 0.71 | 0.53 | 0.93 |

**Supplemental Table 7. Results of Association Studies in the Brazilian (Rio de Janeiro) Children.**

| **rs#** |  | **Genotype frequency (%)** | | | | | | **p-value** | | | |
| --- | --- | --- | --- | --- | --- | --- | --- | --- | --- | --- | --- |
|  | **Allele** | **Affected group** | | | **Unaffected group** | | | **Genotype** | **Allele** | **Dominant Model** | **Recessive Model** |
|  |  | **dd** | **dD** | **DD** | **dd** | **dD** | **DD** |  |  |  |  |
| rs4903399 | T/C | 4 | 52 | 143 | 9 | 88 | 229 | 0.83 | 0.61 | 0.69 | 0.59 |
| rs6547293 | A/G | 2 | 41 | 158 | 4 | 75 | 262 | 0.89 | 0.63 | 0.63 | 0.85 |
| rs1077430 | C/T | 21 | 83 | 94 | 42 | 125 | 146 | 0.63 | 0.54 | 0.85 | 0.35 |
| rs2860216 | C/T | 18 | 87 | 109 | 37 | 14 | 176 | 0.73 | 0.54 | 0.75 | 0.43 |
| rs10132091 | C/T | 37 | 97 | 80 | 51 | 177 | 125 | 0.48 | 0.88 | 0.63 | 0.36 |
| rs1676303 | C/T | 10 | 62 | 142 | 14 | 109 | 231 | 0.85 | 0.94 | 0.79 | 0.68 |
| rs745011 | C/T | 47 | 73 | 78 | 77 | 145 | 126 | 0.54 | 0.8 | 0.46 | 0.66 |

**Supplemental Table 8. Results of Association Studies in the Argentinean Population.**

| **rs#** |  | **Genotype frequency (%)** | | | | | | **p-value** | | | |
| --- | --- | --- | --- | --- | --- | --- | --- | --- | --- | --- | --- |
|  | **Allele** | **Affected group** | | | **Unaffected group** | | | **Genotype** | **Allele** | **Dominant Model** | **Recessive Model** |
|  |  | **dd** | **dD** | **DD** | **dd** | **dD** | **DD** |  |  |  |  |
| rs4903399 | T/C | 2 | 20 | 31 | 1 | 10 | 11 | 0.8 | 0.54 | 0.5 | 0.88 |
| rs6547293 | A/G | 1 | 12 | 49 | 0 | 6 | 18 | 0.71 | 0.82 | 0.69 | 0.53 |
| rs1077430 | C/T | 8 | 15 | 14 | 1 | 8 | 6 | 0.41 | 0.42 | 0.88 | 0.2 |
| rs2860216 | C/T | 13 | 27 | 18 | 8 | 9 | 8 | 0.58 | 0.61 | 0.93 | 0.36 |
| rs10132091 | C/T | 3 | 18 | 17 | 1 | 9 | 9 | 0.93 | 0.77 | 0.85 | 0.71 |
| rs1676303 | C/T | 0 | 3 | 33 | 0 | 2 | 11 | NA | 0.48 | 0.47 | NA |
| rs745011 | C/T | 7 | 18 | 33 | 3 | 11 | 10 | 0.41 | 0.32 | 0.21 | 0.96 |

**Supplemental Table 9. Summary of Sequencing Results and Case-Control Comparisons.**

| **Allele** | **SNP** | **Homozygous** | **Heterozygous** | **Homozygous** |
| --- | --- | --- | --- | --- |
| T/C | rs10132091 | **TT-**22 (27.5%) | **CT-**44 (55.0%) | **CC-**14 (17.5%) |
| C/T | rs61742642 | **CC-**71 (87.7%) | **CT-**9 (11.3%) | **TT-**0 (0.0%) |
| T/C | rs3813545 | **TT-**53 (71.6%) | **CT-**20 (27.0%) | **CC-**1 (1.4%) |
| T/C | rs3829784 | **TT-**10 (14.1%) | **CT-**43 (60.5%) | **CC-**18 (25.4%) |
| G/C | rs45533334 | **GG-**52 (91.2%) | **CG-**4 (7.0%) | **CC-**1 (1.8%) |
| C/A | rs35544003 | **CC-**69 (92%) | **CA-**6 (8.0%) | **AA-**0 (0.0%) |
| C/G | rs2361292 | **CC-**11 (14.1%) | **CG-**27 (34.6%) | **GG-**40 (51.3%) |
| T/C | rs55835922 | **TT-**40 (54.8%) | **CT-**18 (24.7%) | **CC-**15 (20.5%) |
|  |  | **Caries Affected Frequencies** | | |
| **Allele** | **SNP** | **Homozygous** | **Heterozygous** | **Homozygous** |
| T/C | rs10132091 | **TT-**16 (30.2%) | **CT-**27 (50.9%) | **CC-**10 (18.9%) |
| C/T | rs61742642 | **CC-**40 (86.9%) | **CT-**6 (13.0%) | **TT-**0 (0.0%) |
| T/C | rs3813545 | **TT-**37 (74.0%) | **CT-**12 (24.0%) | **CC-**1 (2.0%) |
| T/C | rs3829784 | **TT-**7 (15.2%) | **CT-**27 (58.7%) | **CC-**12 (24.0%) |
| G/C | rs45533334 | **GG-**34 (87.2%) | **CG-**4 (10.3%) | **CC-**1 (2.6%) |
| C/A | rs35544003 | **CC-**48 (90.6%) | **CA-**5 (9.4%) | **AA-**0 (0.0%) |
| C/G | rs2361292 | **CC-**9 (16.7%) | **CG-**16 (29.6%) | **GG-**29 (53.7%) |
| T/C | rs55835922 | **TT-**28 (59.6%) | **CT-**14 (29.8%) | **CC-**5 (10.6%) |
|  |  | **Caries-Free Frequencies** | | |
| **Allele** | **SNP** | **Homozygous** | **Heterozygous** | **Homozygous** |
| T/C | rs10132091 | **TT-**6 (22.2%) | **CT-**17 (62.9%) | **CC-**4 (14.8%) |
| C/T | rs61742642 | **CC-**31 (91.2%) | **CT-**3 (8.8%) | **TT-**0 (0.0%) |
| T/C | rs3813545 | **TT-**16 (66.7%) | **CT-**8 (33.3%) | **CC-**0 (0.0%) |
| T/C | rs3829784 | **TT-**3 (12.0%) | **CT-**16 (64.0%) | **CC-**6 (24.0%) |
| G/C | rs45533334 | **GG-**18 (100%) | **CG-**0 (0.0%) | **CC-**0 (0.0%) |
| C/A | rs35544003 | **CC-**21 (95.5%) | **CA-**1 (4.5%) | **AA-**0 (0.0%) |
| C/G | rs2361292 | **CC-**2 (8.3%) | **CG-**11 (45.8%) | **GG-**11 (45.8%) |

**Supplemental Table 10. Detailed Methods and Results of Enamel Microhardness Testing.**

| **rs#** | **Tooth Group** |  | **Genotype frequency (%)** | | | | | | **p-value** | | | |
| --- | --- | --- | --- | --- | --- | --- | --- | --- | --- | --- | --- | --- |
|  |  | **Tooth Surface** | **Above Mean Enamel Microhardness** | | | **Below Mean Enamel Microhardness** | | | **Genotype** | **Allele** | **Dominant Model** | **Recessive Model** |
|  |  |  | **dd** | **dD** | **DD** | **dd** | **dD** | **DD** |  |  |  |  |
| rs4903399 | Baseline Teeth | Mesial | 7 | 22 | 29 | 2 | 11 | 18 | 0.6339 | 0.3362 | 0.4678 | 0.4023 |
| rs6574293 | Baseline Teeth | Mesial | 1 | 3 | 53 | 1 | 2 | 34 | 0.09857 | 0.1023 | 0.844 | 0.7556 |
| rs4903419 | Baseline Teeth | Mesial | 3 | 38 | 20 | 8 | 19 | 11 | 0.04481 | 0.1628 | 0.6687 | 0.01298 |
| rs1077430 | Baseline Teeth | Mesial | 4 | 16 | 20 | 3 | 3 | 14 | 0.1453 | 0.3857 | 0.1405 | 0.5695 |
| rs2860216 | Baseline Teeth | Mesial | 7 | 25 | 30 | 2 | 18 | 18 | 0.5412 | 0.7089 | 0.9212 | 0.3067 |
| rs10132091 | Baseline Teeth | Mesial | 14 | 26 | 22 | 9 | 19 | 10 | 0.6515 | 0.479 | 0.3401 | 0.8987 |
| rs1676303 | Baseline Teeth | Mesial | 1 | 11 | 50 | 2 | 14 | 22 | 0.04555 | 0.01229 | 0.01392 | 0.299 |
| rs745011 | Baseline Teeth | Mesial | 11 | 21 | 26 | 9 | 13 | 12 | 0.5913 | 0.2553 | 0.37 | 0.3996 |
| rs4903399 | Baseline Teeth | Distal | 5 | 19 | 28 | 4 | 14 | 19 | 0.9676 | 0.7884 | 0.8163 | 0.8537 |
| rs6574293 | Baseline Teeth | Distal | 1 | 4 | 50 | 1 | 1 | 37 | 0.5917 | 0.6108 | 0.4709 | 0.805 |
| rs4903419 | Baseline Teeth | Distal | 7 | 35 | 17 | 4 | 22 | 14 | 0.8012 | 0.5703 | 0.5149 | 0.7721 |
| rs1077430 | Baseline Teeth | Distal | 3 | 10 | 20 | 4 | 9 | 14 | 0.7186 | 0.3769 | 0.496 | 0.492 |
| rs2860216 | Baseline Teeth | Distal | 6 | 26 | 27 | 3 | 17 | 21 | 0.8159 | 0.5302 | 0.5911 | 0.624 |
| rs10132091 | Baseline Teeth | Distal | 15 | 27 | 17 | 8 | 18 | 15 | 0.6561 | 0.3393 | 0.4125 | 0.4896 |
| rs1676303 | Baseline Teeth | Distal | 1 | 14 | 44 | 2 | 11 | 28 | 0.5937 | 0.363 | 0.4913 | 0.3587 |
| rs745011 | Baseline Teeth | Distal | 11 | 18 | 26 | 9 | 16 | 12 | 0.3621 | 0.1937 | 0.1563 | 0.622 |
| rs4903399 | Baseline Teeth | Buccal | 6 | 21 | 27 | 3 | 12 | 20 | 0.7938 | 0.4853 | 0.5097 | 0.6979 |
| rs6574293 | Baseline Teeth | Buccal | 2 | 4 | 53 | 0 | 1 | 34 | 0.3774 | 0.09664 | 0.1917 | 0.2709 |
| rs4903419 | Baseline Teeth | Buccal | 4 | 34 | 23 | 7 | 23 | 8 | 0.07682 | 0.04632 | 0.08231 | 0.06775 |
| rs1077430 | Baseline Teeth | Buccal | 4 | 14 | 19 | 3 | 5 | 15 | 0.4269 | 0.4878 | 0.292 | 0.7934 |
| rs2860216 | Baseline Teeth | Buccal | 4 | 27 | 31 | 5 | 16 | 17 | 0.5156 | 0.3722 | 0.6091 | 0.2554 |
| rs10132091 | Baseline Teeth | Buccal | 13 | 31 | 18 | 10 | 14 | 14 | 0.4386 | 0.8653 | 0.4164 | 0.5373 |
| rs1676303 | Baseline Teeth | Buccal | 2 | 17 | 43 | 1 | 8 | 29 | 0.7527 | 0.4737 | 0.4517 | 0.8657 |
| rs745011 | Baseline Teeth | Buccal | 11 | 24 | 24 | 9 | 10 | 14 | 0.5074 | 0.648 | 0.8704 | 0.3359- |
| rs4903399 | Baseline Teeth | Occlusal | 3 | 20 | 30 | 6 | 13 | 17 | 0.2298 | 0.1399 | 0.3842 | 0.09097 |
| rs6574293 | Baseline Teeth | Occlusal | 2 | 1 | 54 | 0 | 4 | 33 | 0.08926 | 0.7491 | 0.3169 | 0.2494 |
| rs4903419 | Baseline Teeth | Occlusal | 4 | 37 | 20 | 7 | 20 | 11 | 0.1884 | 0.2725 | 0.6887 | 0.06775 |
|  |  |  |  |  |  |  |  |  |  |  |  |  |
| **Supplemental Table 10. Detailed Methods and Results of Enamel Microhardness Testing continued.** | | | | | | | | | | | | |
| rs1077430 | Baseline Teeth | Occlusal | 5 | 11 | 21 | 2 | 8 | 13 | 0.8201 | 0.7846 | 0.9857 | 0.5719 |
| rs2860216 | Baseline Teeth | Occlusal | 2 | 28 | 31 | 7 | 15 | 17 | 0.04385 | 0.1009 | 0.4803 | 0.01241 |
| rs10132091 | Baseline Teeth | Occlusal | 14 | 30 | 17 | 9 | 15 | 15 | 0.4863 | 0.4685 | 0.268 | 0.9883 |
| rs1676303 | Baseline Teeth | Occlusal | 1 | 15 | 45 | 2 | 10 | 27 | 0.5934 | 0.4442 | 0.6219 | 0.3185 |
| rs745011 | Baseline Teeth | Occlusal | 13 | 21 | 23 | 7 | 13 | 15 | 0.9455 | 0.7212 | 0.8126 | 0.7513 |
| rs4903399 | Baseline Teeth | Lingual | 6 | 20 | 31 | 3 | 13 | 16 | 0.8733 | 0.8189 | 0.6908 | 0.8627 |
| rs6574293 | Baseline Teeth | Lingual | 1 | 3 | 54 | 1 | 2 | 33 | 0.9385 | 0.6975 | 0.7965 | 0.7308 |
| rs4903419 | Baseline Teeth | Lingual | 8 | 34 | 19 | 3 | 23 | 12 | 0.7158 | 0.6929 | 0.9641 | 0.4216 |
| rs1077430 | Baseline Teeth | Lingual | 3 | 10 | 21 | 4 | 9 | 13 | 0.5977 | 0.2653 | 0.3621 | 0.4327 |
| rs2860216 | Baseline Teeth | Lingual | 7 | 27 | 28 | 2 | 16 | 20 | 0.5389 | 0.3143 | 0.468 | 0.3067 |
| rs10132091 | Baseline Teeth | Lingual | 12 | 29 | 21 | 11 | 16 | 11 | 0.5392 | 0.3171 | 0.6084 | 0.2686 |
| rs1676303 | Baseline Teeth | Lingual | 3 | 13 | 46 | 0 | 12 | 26 | 0.221 | 0.9294 | 0.5326 | 0.1686 |
| rs745011 | Baseline Teeth | Lingual | 12 | 20 | 24 | 8 | 14 | 14 | 0.9279 | 0.7479 | 0.706 | 0.9282 |
| rs4903399 | Baseline Teeth | Tooth | 5 | 21 | 26 | 4 | 12 | 21 | 0.746 | 0.6859 | 0.5291 | 0.8537 |
| rs6574293 | Baseline Teeth | Tooth | 1 | 4 | 49 | 1 | 1 | 38 | 0.5681 | 0.5664 | 0.4367 | 0.8295 |
| rs4903419 | Baseline Teeth | Tooth | 1 | 35 | 20 | 10 | 22 | 11 | 0.003294 | 0.02441 | 0.2812 | 0.0007535 |
| rs1077430 | Baseline Teeth | Tooth | 3 | 12 | 16 | 4 | 7 | 18 | 0.4697 | 0.6975 | 0.4141 | 0.6197 |
| rs2860216 | Baseline Teeth | Tooth | 3 | 25 | 29 | 6 | 18 | 19 | 0.3154 | 0.2422 | 0.5073 | 0.1327 |
| rs10132091 | Baseline Teeth | Tooth | 11 | 29 | 17 | 12 | 16 | 15 | 0.3673 | 0.8029 | 0.5913 | 0.3112 |
| rs1676303 | Baseline Teeth | Tooth | 0 | 12 | 45 | 3 | 13 | 27 | 0.05809 | 0.02524 | 0.07484 | 0.04289 |
| rs745011 | Baseline Teeth | Tooth | 12 | 21 | 20 | 8 | 13 | 18 | 0.7144 | 0.471 | 0.4177 | 0.8067 |
| rs4903399 | Artificial Caries | Mesial | 7 | 16 | 25 | 2 | 17 | 22 | 0.2917 | 0.4068 | 0.882 | 0.1301 |
| rs6574293 | Artificial Caries | Mesial | 0 | 3 | 45 | 2 | 2 | 42 | 0.3227 | 0.2755 | 0.6516 | 0.1442 |
| rs4903419 | Artificial Caries | Mesial | 3 | 29 | 18 | 8 | 28 | 13 | 0.2136 | 0.155 | 0.3097 | 0.1021 |
| rs1077430 | Artificial Caries | Mesial | 3 | 11 | 15 | 4 | 8 | 19 | 0.6 | 0.6675 | 0.4549 | 0.7577 |
| rs2860216 | Artificial Caries | Mesial | 6 | 24 | 21 | 3 | 19 | 27 | 0.3179 | 0.133 | 0.1635 | 0.3243 |
| rs10132091 | Artificial Caries | Mesial | 10 | 21 | 20 | 13 | 24 | 12 | 0.2791 | 0.1244 | 0.1145 | 0.4109 |
| rs1676303 | Artificial Caries | Mesial | 1 | 10 | 40 | 2 | 15 | 32 | 0.3357 | 0.1365 | 0.1439 | 0.5343 |
| rs745011 | Artificial Caries | Mesial | 10 | 19 | 18 | 10 | 15 | 20 | 0.7662 | 0.7191 | 0.5495 | 0.9125 |
| rs4903399 | Artificial Caries | Distal | 3 | 15 | 22 | 6 | 18 | 25 | 0.7558 | 0.522 | 0.7083 | 0.4602 |
| rs6574293 | Artificial Caries | Distal | 1 | 4 | 40 | 1 | 1 | 47 | 0.3334 | 0.2474 | 0.1947 | 0.9515 |
|  |  |  |  |  |  |  |  |  |  |  |  |  |
| **Supplemental Table 10. Detailed Methods and Results of Enamel Microhardness Testing continued.** | | | | | | | | | | | | |
| rs4903419 | Artificial Caries | Distal | 5 | 26 | 15 | 6 | 31 | 16 | 0.967 | 0.837 | 0.7957 | 0.9432 |
| rs1077430 | Artificial Caries | Distal | 2 | 11 | 21 | 5 | 8 | 13 | 0.2696 | 0.1269 | 0.3621 | 0.1105 |
| rs2860216 | Artificial Caries | Distal | 7 | 17 | 23 | 2 | 26 | 25 | 0.1108 | 0.4734 | 0.8599 | 0.05246 |
| rs10132091 | Artificial Caries | Distal | 11 | 20 | 16 | 12 | 25 | 16 | 0.887 | 0.8266 | 0.6801 | 0.9279 |
| rs1676303 | Artificial Caries | Distal | 1 | 9 | 37 | 2 | 16 | 35 | 0.3686 | 0.1622 | 0.1585 | 0.6301 |
| rs745011 | Artificial Caries | Distal | 8 | 15 | 20 | 12 | 19 | 18 | 0.61 | 0.2797 | 0.342 | 0.4947 |
| rs4903399 | Artificial Caries | Buccal | 6 | 21 | 23 | 3 | 12 | 24 | 0.3415 | 0.1463 | 0.1451 | 0.5036 |
| rs6574293 | Artificial Caries | Buccal | 2 | 2 | 46 | 0 | 3 | 41 | 0.3477 | 0.4064 | 0.8276 | 0.1799 |
| rs4903419 | Artificial Caries | Buccal | 4 | 31 | 19 | 7 | 26 | 12 | 0.3613 | 0.2331 | 0.3628 | 0.199 |
| rs1077430 | Artificial Caries | Buccal | 6 | 13 | 17 | 1 | 6 | 17 | 0.1418 | 0.03 | 0.07059 | 0.1395 |
| rs2860216 | Artificial Caries | Buccal | 4 | 25 | 26 | 5 | 18 | 22 | 0.7446 | 0.8652 | 0.8721 | 0.5046 |
| rs10132091 | Artificial Caries | Buccal | 9 | 25 | 21 | 14 | 20 | 11 | 0.1492 | 0.04419 | 0.1429 | 0.08126 |
| rs1676303 | Artificial Caries | Buccal | 0 | 17 | 38 | 3 | 8 | 34 | 0.06337 | 0.9843 | 0.4738 | 0.05187 |
| rs745011 | Artificial Caries | Buccal | 12 | 18 | 22 | 8 | 16 | 16 | 0.8586 | 0.9579 | 0.8237 | 0.7228 |
| rs4903399 | Artificial Caries | Occlusal | 4 | 19 | 19 | 5 | 14 | 28 | 0.3137 | 0.3301 | 0.1762 | 0.8618 |
| rs6574293 | Artificial Caries | Occlusal | 1 | 3 | 37 | 1 | 2 | 50 | 0.7332 | 0.4592 | 0.4532 | 0.854 |
| rs4903419 | Artificial Caries | Occlusal | 0 | 28 | 16 | 11 | 29 | 15 | 0.006097 | 0.03781 | 0.3325 | 0.001653 |
| rs1077430 | Artificial Caries | Occlusal | 3 | 6 | 17 | 4 | 13 | 17 | 0.4306 | 0.3427 | 0.2334 | 0.9784 |
| rs2860216 | Artificial Caries | Occlusal | 3 | 17 | 25 | 6 | 26 | 23 | 0.3703 | 0.1695 | 0.1713 | 0.4608 |
| rs10132091 | Artificial Caries | Occlusal | 8 | 21 | 16 | 15 | 24 | 16 | 0.5107 | 0.2596 | 0.4905 | 0.2617 |
| rs1676303 | Artificial Caries | Occlusal | 0 | 12 | 33 | 3 | 13 | 39 | 0.2773 | 0.4438 | 0.7882 | 0.1117 |
| rs745011 | Artificial Caries | Occlusal | 9 | 16 | 17 | 11 | 18 | 21 | 0.9785 | 0.9477 | 0.8825 | 0.9472 |
| rs4903399 | Artificial Caries | Lingual | 4 | 17 | 22 | 5 | 16 | 25 | 0.8905 | 0.9051 | 0.7636 | 0.8064 |
| rs6574293 | Artificial Caries | Lingual | 0 | 4 | 39 | 2 | 1 | 48 | 0.13 | 0.936 | 0.5292 | 0.1893 |
| rs4903419 | Artificial Caries | Lingual | 4 | 32 | 12 | 7 | 25 | 19 | 0.2049 | 0.6222 | 0.1888 | 0.3936 |
| rs1077430 | Artificial Caries | Lingual | 3 | 10 | 14 | 4 | 9 | 20 | 0.7186 | 0.6365 | 0.496 | 0.9035 |
| rs2860216 | Artificial Caries | Lingual | 7 | 20 | 21 | 2 | 23 | 27 | 0.1667 | 0.1468 | 0.4138 | 0.06087 |
| rs10132091 | Artificial Caries | Lingual | 6 | 25 | 17 | 17 | 20 | 15 | 0.05528 | 0.05762 | 0.4816 | 0.01652 |
| rs1676303 | Artificial Caries | Lingual | 1 | 14 | 33 | 2 | 11 | 39 | 0.596 | 0.6614 | 0.4868 | 0.6057 |
| rs745011 | Artificial Caries | Lingual | 8 | 19 | 18 | 12 | 15 | 20 | 0.5135 | 0.7191 | 0.8037 | 0.3674 |
| rs4903399 | Artificial Caries | Tooth | 4 | 20 | 22 | 5 | 13 | 25 | 0.4299 | 0.5863 | 0.3301 | 0.6466 |
|  |  |  |  |  |  |  |  |  |  |  |  |  |
| **Supplemental Table 10. Detailed Methods and Results of Enamel Microhardness Testing continued.** | | | | | | | | | | | | |
| rs6574293 | Artificial Caries | Tooth | 1 | 3 | 42 | 1 | 2 | 45 | 0.8777 | 0.6839 | 0.6516 | 0.9757 |
| rs4903419 | Artificial Caries | Tooth | 2 | 32 | 15 | 9 | 25 | 16 | 0.06936 | 0.3681 | 0.8817 | 0.02758 |
| rs1077430 | Artificial Caries | Tooth | 4 | 10 | 15 | 3 | 9 | 19 | 0.7408 | 0.4016 | 0.4549 | 0.6197 |
| rs2860216 | Artificial Caries | Tooth | 6 | 22 | 22 | 3 | 21 | 26 | 0.5075 | 0.2823 | 0.4233 | 0.2945 |
| rs10132091 | Artificial Caries | Tooth | 9 | 23 | 18 | 14 | 22 | 14 | 0.4473 | 0.2013 | 0.3912 | 0.2348 |
| rs1676303 | Artificial Caries | Tooth | 0 | 13 | 37 | 3 | 12 | 35 | 0.2127 | 0.3286 | 0.656 | 0.07864 |
| rs745011 | Artificial Caries | Tooth | 11 | 17 | 19 | 9 | 17 | 19 | 0.9247 | 0.7191 | 0.8611 | 0.6923 |
| rs4903399 | Fluoride Teeth | Mesial | 6 | 15 | 24 | 3 | 18 | 23 | 0.5265 | 0.6874 | 0.9202 | 0.3081 |
| rs6574293 | Fluoride Teeth | Mesial | 0 | 3 | 43 | 2 | 2 | 44 | 0.3379 | 0.3372 | 0.738 | 0.1617 |
| rs4903419 | Fluoride Teeth | Mesial | 3 | 28 | 17 | 8 | 29 | 14 | 0.2877 | 0.2115 | 0.393 | 0.1354 |
| rs1077430 | Fluoride Teeth | Mesial | 3 | 10 | 12 | 4 | 9 | 20 | 0.7186 | 0.6365 | 0.496 | 0.9035 |
| rs2860216 | Fluoride Teeth | Mesial | 6 | 25 | 18 | 3 | 18 | 30 | 0.07802 | 0.02893 | 0.02709 | 0.2664 |
| rs10132091 | Fluoride Teeth | Mesial | 9 | 21 | 19 | 14 | 24 | 13 | 0.3053 | 0.1123 | 0.1545 | 0.2806 |
| rs1676303 | Fluoride Teeth | Mesial | 1 | 9 | 39 | 2 | 16 | 33 | 0.2523 | 0.1015 | 0.09745 | 0.5815 |
| rs745011 | Fluoride Teeth | Mesial | 10 | 19 | 16 | 10 | 15 | 22 | 0.5028 | 0.399 | 0.2732 | 0.9125 |
| rs4903399 | Fluoride Teeth | Distal | 3 | 16 | 24 | 6 | 17 | 23 | 0.6214 | 0.3811 | 0.583 | 0.3428 |
| rs6574293 | Fluoride Teeth | Distal | 2 | 5 | 41 | 0 | 0 | 46 | 0.02667 | 0.002615 | 0.007098 | 0.1617 |
| rs4903419 | Fluoride Teeth | Distal | 5 | 29 | 15 | 6 | 28 | 16 | 0.9368 | 0.9766 | 0.8817 | 0.7762 |
| rs1077430 | Fluoride Teeth | Distal | 3 | 10 | 21 | 4 | 9 | 13 | 0.5977 | 0.2653 | 0.3621 | 0.4327 |
| rs2860216 | Fluoride Teeth | Distal | 7 | 18 | 25 | 2 | 25 | 23 | 0.1353 | 0.645 | 0.6889 | 0.08061 |
| rs10132091 | Fluoride Teeth | Distal | 11 | 21 | 18 | 12 | 24 | 14 | 0.6895 | 0.4777 | 0.3912 | 0.8122 |
| rs1676303 | Fluoride Teeth | Distal | 2 | 9 | 39 | 1 | 16 | 33 | 0.2474 | 0.3286 | 0.1814 | 0.5577 |
| rs745011 | Fluoride Teeth | Distal | 7 | 18 | 21 | 13 | 16 | 17 | 0.3106 | 0.1327 | 0.397 | 0.1294 |
| rs4903399 | Fluoride Teeth | Buccal | 6 | 23 | 23 | 3 | 10 | 24 | 0.1557 | 0.08014 | 0.05463 | 0.5968 |
| rs6574293 | Fluoride Teeth | Buccal | 2 | 1 | 48 | 0 | 4 | 39 | 0.13 | 0.936 | 0.5292 | 0.1893 |
| rs4903419 | Fluoride Teeth | Buccal | 5 | 34 | 16 | 6 | 23 | 15 | 0.5955 | 0.9741 | 0.594 | 0.4745 |
| rs1077430 | Fluoride Teeth | Buccal | 6 | 14 | 17 | 1 | 5 | 17 | 0.08933 | 0.01751 | 0.03354 | 0.1638 |
| rs2860216 | Fluoride Teeth | Buccal | 5 | 24 | 27 | 4 | 19 | 21 | 0.9987 | 0.9605 | 0.9614 | 0.9775 |
| rs10132091 | Fluoride Teeth | Buccal | 9 | 25 | 22 | 14 | 20 | 10 | 0.09203 | 0.02278 | 0.07807 | 0.06326 |
| rs1676303 | Fluoride Teeth | Buccal | 0 | 14 | 42 | 3 | 11 | 30 | 0.1369 | 0.186 | 0.451 | 0.04726 |
| rs745011 | Fluoride Teeth | Buccal | 12 | 19 | 22 | 8 | 15 | 16 | 0.9562 | 0.9105 | 0.9629 | 0.8067 |
|  |  |  |  |  |  |  |  |  |  |  |  |  |
| **Supplemental Table 10. Detailed Methods and Results of Enamel Microhardness Testing continued.** | | | | | | | | | | | | |
| rs4903399 | Fluoride Teeth | Occlusal | 3 | 18 | 23 | 6 | 15 | 24 | 0.5265 | 0.6874 | 0.9202 | 0.3081 |
| rs6574293 | Fluoride Teeth | Occlusal | 2 | 3 | 39 | 0 | 2 | 48 | 0.2517 | 0.05637 | 0.1748 | 0.1275 |
| rs4903419 | Fluoride Teeth | Occlusal | 1 | 30 | 17 | 10 | 27 | 14 | 0.02098 | 0.0672 | 0.393 | 0.005556 |
| rs1077430 | Fluoride Teeth | Occlusal | 4 | 9 | 16 | 3 | 10 | 18 | 0.8839 | 0.6675 | 0.8213 | 0.6197 |
| rs2860216 | Fluoride Teeth | Occlusal | 3 | 17 | 28 | 6 | 26 | 20 | 0.1311 | 0.05354 | 0.0469 | 0.3559 |
| rs10132091 | Fluoride Teeth | Occlusal | 11 | 21 | 16 | 12 | 24 | 16 | 0.9591 | 0.8467 | 0.7836 | 0.9848 |
| rs1676303 | Fluoride Teeth | Occlusal | 0 | 13 | 35 | 3 | 12 | 37 | 0.2299 | 0.4622 | 0.8445 | 0.0911 |
| rs745011 | Fluoride Teeth | Occlusal | 8 | 18 | 19 | 12 | 16 | 19 | 0.6458 | 0.509 | 0.8611 | 0.3674 |
| rs4903399 | Fluoride Teeth | Lingual | 5 | 17 | 23 | 4 | 16 | 24 | 0.9271 | 0.6874 | 0.7456 | 0.752 |
| rs6574293 | Fluoride Teeth | Lingual | 0 | 4 | 41 | 2 | 1 | 46 | 0.1406 | 0.8329 | 0.6098 | 0.1707 |
| rs4903419 | Fluoride Teeth | Lingual | 5 | 32 | 13 | 6 | 25 | 18 | 0.4175 | 0.542 | 0.2495 | 0.7223 |
| rs1077430 | Fluoride Teeth | Lingual | 3 | 11 | 14 | 4 | 8 | 20 | 0.4929 | 0.512 | 0.3297 | 0.8298 |
| rs2860216 | Fluoride Teeth | Lingual | 7 | 21 | 22 | 2 | 22 | 26 | 0.2086 | 0.1669 | 0.4233 | 0.08061 |
| rs10132091 | Fluoride Teeth | Lingual | 8 | 25 | 17 | 15 | 20 | 15 | 0.2452 | 0.2013 | 0.6681 | 0.09624 |
| rs1676303 | Fluoride Teeth | Lingual | 1 | 14 | 35 | 2 | 11 | 37 | 0.6877 | 0.8451 | 0.656 | 0.5577 |
| rs745011 | Fluoride Teeth | Lingual | 8 | 19 | 20 | 12 | 15 | 18 | 0.5135 | 0.399 | 0.8037 | 0.2622 |
| rs4903399 | Fluoride Teeth | Tooth | 4 | 20 | 20 | 5 | 13 | 27 | 0.2688 | 0.3555 | 0.1693 | 0.752 |
| rs6574293 | Fluoride Teeth | Tooth | 2 | 4 | 39 | 0 | 1 | 48 | 0.1018 | 0.01159 | 0.03722 | 0.1358 |
| rs4903419 | Fluoride Teeth | Tooth | 3 | 32 | 13 | 8 | 25 | 18 | 0.1458 | 0.9299 | 0.3787 | 0.1354 |
| rs1077430 | Fluoride Teeth | Tooth | 5 | 8 | 16 | 2 | 11 | 18 | 0.4041 | 0.4016 | 0.8213 | 0.1933 |
| rs2860216 | Fluoride Teeth | Tooth | 7 | 21 | 21 | 2 | 22 | 27 | 0.1727 | 0.1164 | 0.313 | 0.07023 |
| rs10132091 | Fluoride Teeth | Tooth | 8 | 22 | 19 | 15 | 23 | 13 | 0.198 | 0.06122 | 0.1545 | 0.1201 |
| rs1676303 | Fluoride Teeth | Tooth | 0 | 11 | 38 | 3 | 14 | 34 | 0.17 | 0.1015 | 0.2256 | 0.08474 |
| rs745011 | Fluoride Teeth | Tooth | 8 | 17 | 21 | 12 | 17 | 17 | 0.5431 | 0.2291 | 0.397 | 0.312 |
| rs4903399 | Baseline-Artificial Caries | Mesial | 1 | 19 | 21 | 8 | 14 | 26 | 0.04456 | 0.4068 | 0.7813 | 0.02648 |
| rs6574293 | Baseline-Artificial Caries | Mesial | 1 | 2 | 40 | 1 | 3 | 47 | 0.9594 | 0.936 | 0.8734 | 0.9028 |
| rs4903419 | Baseline-Artificial Caries | Mesial | 4 | 28 | 14 | 7 | 29 | 17 | 0.7282 | 0.837 | 0.8606 | 0.4762 |
| rs1077430 | Baseline-Artificial Caries | Mesial | 3 | 8 | 18 | 4 | 11 | 16 | 0.716 | 0.425 | 0.4141 | 0.7577 |
| rs2860216 | Baseline-Artificial Caries | Mesial | 3 | 17 | 26 | 6 | 26 | 22 | 0.2734 | 0.1189 | 0.1154 | 0.4241 |
| **Supplemental Table 10. Detailed Methods and Results of Enamel Microhardness Testing continued.** | | | | | | | | | | | | |
| rs10132091 | Baseline-Artificial Caries | Mesial | 10 | 23 | 13 | 13 | 22 | 19 | 0.6363 | 0.7453 | 0.4594 | 0.7821 |
| rs1676303 | Baseline-Artificial Caries | Mesial | 1 | 13 | 32 | 2 | 12 | 40 | 0.7312 | 0.7717 | 0.6167 | 0.6549 |
| rs745011 | Baseline-Artificial Caries | Mesial | 10 | 14 | 19 | 10 | 20 | 19 | 0.7152 | 0.8596 | 0.599 | 0.7411 |
| rs4903399 | Baseline-Artificial Caries | Distal | 6 | 18 | 22 | 3 | 15 | 25 | 0.5054 | 0.2272 | 0.3301 | 0.3428 |
| rs6574293 | Baseline-Artificial Caries | Distal | 0 | 3 | 43 | 2 | 2 | 44 | 0.3379 | 0.3372 | 0.738 | 0.1617 |
| rs4903419 | Baseline-Artificial Caries | Distal | 5 | 29 | 15 | 6 | 28 | 16 | 0.9368 | 0.9766 | 0.8817 | 0.7762 |
| rs1077430 | Baseline-Artificial Caries | Distal | 4 | 8 | 12 | 3 | 11 | 22 | 0.5472 | 0.2426 | 0.3948 | 0.3246 |
| rs2860216 | Baseline-Artificial Caries | Distal | 2 | 25 | 22 | 7 | 18 | 26 | 0.1217 | 0.7845 | 0.5428 | 0.09207 |
| rs10132091 | Baseline-Artificial Caries | Distal | 13 | 24 | 12 | 10 | 21 | 20 | 0.2791 | 0.1244 | 0.1145 | 0.4109 |
| rs1676303 | Baseline-Artificial Caries | Distal | 1 | 12 | 36 | 2 | 13 | 36 | 0.8464 | 0.6419 | 0.7484 | 0.5815 |
| rs745011 | Baseline-Artificial Caries | Distal | 3 | 14 | 18 | 7 | 20 | 20 | 0.232 | 0.2525 | 0.8037 | 0.1038 |
| rs4903399 | Baseline-Artificial Caries | Buccal | 2 | 11 | 22 | 7 | 22 | 25 | 0.2589 | 0.08617 | 0.1263 | 0.2679 |
| rs6574293 | Baseline-Artificial Caries | Buccal | 0 | 4 | 36 | 2 | 1 | 51 | 0.1109 | 0.9064 | 0.4171 | 0.2186 |
| rs4903419 | Baseline-Artificial Caries | Buccal | 5 | 23 | 13 | 6 | 34 | 18 | 0.9493 | 0.9336 | 0.9433 | 0.7729 |
| rs1077430 | Baseline-Artificial Caries | Buccal | 1 | 5 | 15 | 6 | 14 | 19 | 0.2027 | 0.05114 | 0.09041 | 0.2215 |
| rs2860216 | Baseline-Artificial Caries | Buccal | 3 | 18 | 20 | 6 | 25 | 28 | 0.8866 | 0.7525 | 0.8964 | 0.624 |
| rs10132091 | Baseline-Artificial Caries | Buccal | 9 | 24 | 8 | 14 | 21 | 24 | 0.04395 | 0.1757 | 0.02564 | 0.8354 |
| rs1676303 | Baseline-Artificial Caries | Buccal | 3 | 9 | 29 | 0 | 16 | 43 | 0.1007 | 0.363 | 0.8138 | 0.03489 |
| rs745011 | Baseline-Artificial Caries | Buccal | 8 | 15 | 13 | 12 | 19 | 25 | 0.6881 | 0.529 | 0.4173 | 0.9282 |
| rs4903399 | Baseline-Artificial Caries | Occlusal | 3 | 15 | 20 | 6 | 18 | 27 | 0.8084 | 0.795 | 0.9769 | 0.5492 |
| rs6574293 | Baseline-Artificial Caries | Occlusal | 1 | 2 | 41 | 1 | 3 | 46 | 0.9489 | 0.8842 | 0.8276 | 0.9271 |
| **Supplemental Table 10. Detailed Methods and Results of Enamel Microhardness Testing continued.** | | | | | | | | | | | | |
| rs4903419 | Baseline-Artificial Caries | Occlusal | 6 | 25 | 14 | 5 | 32 | 17 | 0.8081 | 0.7505 | 0.9684 | 0.5207 |
| rs1077430 | Baseline-Artificial Caries | Occlusal | 2 | 11 | 15 | 5 | 8 | 19 | 0.3731 | 0.8698 | 0.6508 | 0.3072 |
| rs2860216 | Baseline-Artificial Caries | Occlusal | 3 | 22 | 20 | 6 | 21 | 28 | 0.504 | 0.8652 | 0.5197 | 0.4608 |
| rs10132091 | Baseline-Artificial Caries | Occlusal | 11 | 22 | 12 | 12 | 23 | 20 | 0.5838 | 0.384 | 0.3011 | 0.7562 |
| rs1676303 | Baseline-Artificial Caries | Occlusal | 2 | 11 | 32 | 1 | 14 | 40 | 0.7452 | 0.6801 | 0.8579 | 0.4437 |
| rs745011 | Baseline-Artificial Caries | Occlusal | 10 | 15 | 17 | 10 | 19 | 21 | 0.906 | 0.7133 | 0.8825 | 0.659 |
| rs4903399 | Baseline-Artificial Caries | Lingual | 5 | 15 | 26 | 4 | 18 | 21 | 0.6651 | 0.652 | 0.468 | 0.8064 |
| rs6574293 | Baseline-Artificial Caries | Lingual | 2 | 1 | 46 | 0 | 4 | 41 | 0.1406 | 0.8329 | 0.6098 | 0.1707 |
| rs4903419 | Baseline-Artificial Caries | Lingual | 6 | 25 | 17 | 5 | 32 | 14 | 0.5624 | 0.7051 | 0.393 | 0.6697 |
| rs1077430 | Baseline-Artificial Caries | Lingual | 3 | 10 | 18 | 4 | 9 | 16 | 0.8839 | 0.6675 | 0.8213 | 0.6197 |
| rs2860216 | Baseline-Artificial Caries | Lingual | 2 | 24 | 23 | 7 | 19 | 25 | 0.1823 | 0.5615 | 0.8351 | 0.09207 |
| rs10132091 | Baseline-Artificial Caries | Lingual | 15 | 19 | 15 | 8 | 26 | 17 | 0.1915 | 0.2103 | 0.7706 | 0.07622 |
| rs1676303 | Baseline-Artificial Caries | Lingual | 2 | 8 | 39 | 1 | 17 | 33 | 0.133 | 0.2125 | 0.09745 | 0.5343 |
| rs745011 | Baseline-Artificial Caries | Lingual | 10 | 17 | 18 | 10 | 17 | 20 | 0.9696 | 0.8088 | 0.8037 | 0.9125 |
| rs4903399 | Baseline-Artificial Caries | Tooth | 4 | 16 | 23 | 5 | 17 | 24 | 0.9697 | 0.8317 | 0.9012 | 0.8064 |
| rs6574293 | Baseline-Artificial Caries | Tooth | 0 | 2 | 45 | 2 | 3 | 42 | 0.3161 | 0.08763 | 0.2385 | 0.1529 |
| rs4903419 | Baseline-Artificial Caries | Tooth | 5 | 27 | 17 | 6 | 30 | 14 | 0.7676 | 0.542 | 0.4727 | 0.7762 |
| rs1077430 | Baseline-Artificial Caries | Tooth | 1 | 10 | 16 | 6 | 9 | 18 | 0.2046 | 0.2415 | 0.7139 | 0.08222 |
| rs2860216 | Baseline-Artificial Caries | Tooth | 1 | 23 | 25 | 8 | 20 | 23 | 0.05786 | 0.133 | 0.5535 | 0.01715 |
| rs10132091 | Baseline-Artificial Caries | Tooth | 11 | 27 | 11 | 12 | 18 | 21 | 0.08499 | 0.2103 | 0.04476 | 0.8979 |
| rs1676303 | Baseline-Artificial Caries | Tooth | 2 | 12 | 35 | 1 | 13 | 37 | 0.8232 | 0.7516 | 0.9007 | 0.5343 |
| **Supplemental Table 10. Detailed Methods and Results of Enamel Microhardness Testing continued.** | | | | | | | | | | | | |
| rs745011 | Baseline-Artificial Caries | Tooth | 9 | 18 | 17 | 11 | 16 | 21 | 0.7536 | 0.8546 | 0.6188 | 0.7749 |
| rs4903399 | Fluoride-Artificial Caries | Mesial | 4 | 18 | 21 | 5 | 15 | 26 | 0.6651 | 0.652 | 0.468 | 0.8064 |
| rs6574293 | Fluoride-Artificial Caries | Mesial | 0 | 1 | 42 | 2 | 4 | 45 | 0.1973 | 0.03257 | 0.08245 | 0.1893 |
| rs4903419 | Fluoride-Artificial Caries | Mesial | 8 | 25 | 13 | 3 | 32 | 18 | 0.1772 | 0.2116 | 0.5418 | 0.06397 |
| rs1077430 | Fluoride-Artificial Caries | Mesial | 4 | 10 | 14 | 3 | 9 | 20 | 0.6089 | 0.2867 | 0.3297 | 0.5544 |
| rs2860216 | Fluoride-Artificial Caries | Mesial | 4 | 26 | 16 | 5 | 17 | 32 | 0.03454 | 0.06719 | 0.01462 | 0.9218 |
| rs10132091 | Fluoride-Artificial Caries | Mesail | 8 | 24 | 14 | 15 | 21 | 18 | 0.3321 | 0.5962 | 0.7568 | 0.2187 |
| rs1676303 | Fluoride-Artificial Caries | Mesial | 0 | 11 | 35 | 3 | 14 | 37 | 0.2474 | 0.2012 | 0.4008 | 0.1046 |
| rs745011 | Fluoride-Artificial Caries | Mesial | 9 | 17 | 14 | 11 | 17 | 24 | 0.5251 | 0.3914 | 0.2814 | 0.8767 |
| rs4903399 | Fluoride-Artificial Caries | Distal | 3 | 13 | 21 | 6 | 20 | 26 | 0.7776 | 0.4588 | 0.5291 | 0.5968 |
| rs6574293 | Fluoride-Artificial Caries | Distal | 2 | 5 | 35 | 0 | 0 | 52 | 0.009261 | 0.000624 | 0.002213 | 0.1117 |
| rs4903419 | Fluoride-Artificial Caries | Distal | 3 | 26 | 12 | 8 | 31 | 19 | 0.493 | 0.8327 | 0.7122 | 0.3125 |
| rs1077430 | Fluoride-Artificial Caries | Distal | 5 | 6 | 16 | 2 | 13 | 18 | 0.1812 | 0.6365 | 0.7139 | 0.1348 |
| rs2860216 | Fluoride-Artificial Caries | Distal | 3 | 17 | 22 | 6 | 26 | 26 | 0.7138 | 0.4149 | 0.4555 | 0.5808 |
| rs10132091 | Fluoride-Artificial Caries | Distal | 11 | 18 | 13 | 12 | 27 | 19 | 0.8109 | 0.6086 | 0.8484 | 0.5188 |
| rs1676303 | Fluoride-Artificial Caries | Distal | 1 | 11 | 30 | 2 | 14 | 42 | 0.9339 | 0.9937 | 0.9138 | 0.7575 |
| rs745011 | Fluoride-Artificial Caries | Distal | 8 | 15 | 14 | 12 | 19 | 24 | 0.8205 | 0.704 | 0.5797 | 0.9821 |
| rs4903399 | Fluoride-Artificial Caries | Buccal | 2 | 20 | 14 | 7 | 13 | 33 | 0.01097 | 0.2549 | 0.03016 | 0.2399 |
| rs6574293 | Fluoride-Artificial Caries | Buccal | 2 | 1 | 36 | 0 | 4 | 51 | 0.1517 | 0.3801 | 0.9391 | 0.08958 |
| rs4903419 | Fluoride-Artificial Caries | Buccal | 5 | 26 | 10 | 6 | 31 | 21 | 0.4585 | 0.334 | 0.2117 | 0.7729 |
| rs1077430 | Fluoride-Artificial Caries | Buccal | 3 | 5 | 13 | 4 | 14 | 21 | 0.6138 | 0.8136 | 0.548 | 0.6428 |
| **Supplemental Table 10. Detailed Methods and Results of Enamel Microhardness Testing continued.** | | | | | | | | | | | | |
| rs2860216 | Fluoride-Artificial Caries | Buccal | 4 | 17 | 20 | 5 | 26 | 28 | 0.955 | 0.9975 | 0.8964 | 0.8257 |
| rs10132091 | Fluoride-Artificial Caries | Buccal | 9 | 18 | 14 | 14 | 27 | 18 | 0.9269 | 0.7053 | 0.7013 | 0.8354 |
| rs1676303 | Fluoride-Artificial Caries | Buccal | 2 | 9 | 30 | 1 | 16 | 42 | 0.5802 | 0.9083 | 0.8279 | 0.3587 |
| rs745011 | Fluoride-Artificial Caries | Buccal | 8 | 11 | 16 | 12 | 23 | 22 | 0.682 | 0.7212 | 0.5008 | 0.8386 |
| rs4903399 | Fluoride-Artificial Caries | Occlusal | 3 | 17 | 23 | 6 | 16 | 24 | 0.6214 | 0.5863 | 0.9012 | 0.3428 |
| rs6574293 | Fluoride-Artificial Caries | Occlusal | 1 | 2 | 41 | 1 | 3 | 46 | 0.9489 | 0.8842 | 0.8276 | 0.9271 |
| rs4903419 | Fluoride-Artificial Caries | Occlusal | 6 | 21 | 20 | 5 | 36 | 11 | 0.04046 | 0.1904 | 0.02187 | 0.6184 |
| rs1077430 | Fluoride-Artificial Caries | Occlusal | 4 | 9 | 15 | 3 | 10 | 19 | 0.8182 | 0.512 | 0.6508 | 0.5544 |
| rs2860216 | Fluoride-Artificial Caries | Occlusal | 3 | 20 | 24 | 6 | 23 | 24 | 0.653 | 0.4113 | 0.5636 | 0.3892 |
| rs10132091 | Fluoride-Artificial Caries | Occlusal | 13 | 17 | 17 | 10 | 28 | 15 | 0.2399 | 0.9478 | 0.3999 | 0.2971 |
| rs1676303 | Fluoride-Artificial Caries | Occlusal | 1 | 14 | 32 | 2 | 11 | 40 | 0.5416 | 0.5756 | 0.4116 | 0.6301 |
| rs745011 | Fluoride-Artificial Caries | Occlusal | 9 | 18 | 15 | 11 | 16 | 23 | 0.5178 | 0.5033 | 0.3183 | 0.9472 |
| rs4903399 | Fluoride-Artificial Caries | Lingual | 2 | 14 | 26 | 7 | 19 | 21 | 0.1497 | 0.04392 | 0.1042 | 0.1135 |
| rs6574293 | Fluoride-Artificial Caries | Lingual | 1 | 2 | 41 | 1 | 3 | 46 | 0.9489 | 0.8842 | 0.8276 | 0.9271 |
| rs4903419 | Fluoride-Artificial Caries | Lingual | 9 | 24 | 15 | 2 | 33 | 16 | 0.05441 | 0.283 | 0.9895 | 0.01896 |
| rs1077430 | Fluoride-Artificial Caries | Lingual | 4 | 7 | 18 | 3 | 12 | 16 | 0.4697 | 0.6975 | 0.4141 | 0.6197 |
| rs2860216 | Fluoride-Artificial Caries | Lingual | 7 | 18 | 23 | 2 | 25 | 25 | 0.1461 | 0.4031 | 0.9872 | 0.06087 |
| rs10132091 | Fluoride-Artificial Caries | Lingual | 13 | 22 | 13 | 10 | 23 | 19 | 0.5014 | 0.2195 | 0.3112 | 0.3512 |
| rs1676303 | Fluoride-Artificial Caries | Lingual | 1 | 11 | 36 | 2 | 14 | 36 | 0.7656 | 0.4622 | 0.5209 | 0.6057 |
| rs745011 | Fluoride-Artificial Caries | Lingual | 10 | 13 | 21 | 10 | 21 | 17 | 0.3441 | 0.4717 | 0.2309 | 0.8259 |
| rs4903399 | Fluoride-Artificial Caries | Tooth | 2 | 18 | 24 | 7 | 15 | 23 | 0.2164 | 0.2866 | 0.7456 | 0.08498 |
| **Supplemental Table 10. Detailed Methods and Results of Enamel Microhardness Testing continued.** | | | | | | | | | | | | |
| rs6574293 | Fluoride-Artificial Caries | Tooth | 1 | 4 | 42 | 1 | 1 | 45 | 0.3861 | 0.3054 | 0.2385 | 1 |
| rs4903419 | Fluoride-Artificial Caries | Tooth | 10 | 27 | 13 | 1 | 30 | 18 | 0.01562 | 0.03928 | 0.2495 | 0.004472 |
| rs1077430 | Fluoride-Artificial Caries | Tooth | 1 | 8 | 19 | 6 | 11 | 15 | 0.1184 | 0.0269 | 0.1018 | 0.06768 |
| rs2860216 | Fluoride-Artificial Caries | Tooth | 6 | 21 | 23 | 3 | 22 | 25 | 0.5751 | 0.4425 | 0.6889 | 0.2945 |
| rs10132091 | Fluoride-Artificial Caries | Tooth | 13 | 22 | 15 | 10 | 23 | 17 | 0.7639 | 0.4777 | 0.6681 | 0.4759 |
| rs1676303 | Fluoride-Artificial Caries | Tooth | 3 | 8 | 39 | 0 | 17 | 33 | 0.03439 | 0.5578 | 0.1814 | 0.07864 |
| rs745011 | Fluoride-Artificial Caries | Tooth | 7 | 17 | 19 | 13 | 17 | 19 | 0.4929 | 0.2797 | 0.599 | 0.2343 |
